# N-acetylcysteine during critical neurodevelopmental periods prevents behavioral and neurochemical deficits in the Poly I:C rat model of schizophrenia

**DOI:** 10.1038/s41398-023-02652-7

**Published:** 2024-01-08

**Authors:** Diego Romero-Miguel, Marta Casquero-Veiga, Nicolás Lamanna-Rama, Sonia Torres-Sánchez, Karina S. MacDowell, José A. García-Partida, Cristina Santa-Marta, Esther Berrocoso, Juan C. Leza, Manuel Desco, María Luisa Soto-Montenegro

**Affiliations:** 1 Instituto de Investigación Sanitaria Gregorio Marañón, Madrid, 28007 Spain; 2 Department of Bioengineering, Universidad Carlos III de Madrid, Leganés (Madrid), 28911 Spain; 3 CIBER de Salud Mental (CIBERSAM), Madrid, 28029 Spain; 4 Neuropsychopharmacology & Psychobiology Research Group, Department of Neuroscience, Universidad de Cádiz, Cádiz, 11003 Spain; 5 Instituto de Investigación e Innovación en Ciencias Biomédicas de Cádiz (INiBICA), Hospital Universitario Puerta del Mar, Cádiz, 11009 Spain; 6 Department of Pharmacology & Toxicology, School of Medicine, Universidad Complutense (UCM), IIS Imas12, IUIN, Madrid, 28040 Spain; 7 Department of Mathematical and Fluids Physics, UNED, Madrid, 28040 Spain; 8 Advanced Imaging Unit, Centro Nacional de Investigaciones Cardiovasculares (CNIC), Madrid, 28029 Spain; 9 Grupo de Fisiopatología y Farmacología del Sistema Digestivo de la Universidad Rey Juan Carlos (NeuGut), Alcorcón (Madrid), 28922 Spain; 10 Present Address: Instituto de Investigación Sanitaria Fundación Jiménez Díaz, IIS-FJD, 28040 Madrid, Spain; 11 Present Address: Cardiovascular Imaging and Population Studies, Centro Nacional de Investigaciones Cardiovasculares (CNIC), 28029 Madrid, Spain

**Keywords:** Schizophrenia, Molecular neuroscience

## Abstract

Schizophrenia is a chronic neurodevelopmental disorder with an inflammatory/prooxidant component. N-acetylcysteine (NAC) has been evaluated in schizophrenia as an adjuvant to antipsychotics, but its role as a preventive strategy has not been sufficiently explored. We aimed to evaluate the potential of NAC administration in two-time windows before the onset of symptoms in a schizophrenia-like maternal immune stimulation (MIS) rat model. Pregnant Wistar rats were injected with Poly I:C or Saline on gestational day (GD) 15. Three different preventive approaches were evaluated: 1) NAC treatment during periadolescence in the offspring (from postnatal day [PND] 35 to 49); 2) NAC treatment during pregnancy after MIS challenge until delivery (GD15–21); and 3) NAC treatment throughout all pregnancy (GD1–21). At postnatal day (PND) 70, prepulse inhibition (PPI) and anxiety levels were evaluated. In vivo magnetic resonance (MR) imaging was acquired on PND100 to assess structural changes in gray and white matter, and brain metabolite concentrations. Additionally, inflammation and oxidative stress (IOS) markers were measured ex vivo in selected brain regions. MIS offspring showed behavioral, neuroanatomical, and biochemical alterations. Interestingly, NAC treatment during periadolescence prevented PPI deficits and partially counteracted some biochemical imbalances. Moreover, NAC treatments during pregnancy not only replicated the beneficial outcomes reported by the treatment in periadolescence, but also prevented some neuroanatomical deficits, including reductions in hippocampal and corpus callosum volumes. This study suggests that early reduction of inflammation and prooxidation could help prevent the onset of schizophrenia-like symptoms, supporting the importance of anti-IOS compounds in ameliorating this disorder.

## Introduction

Schizophrenia is a chronic neuropsychiatric disorder that affects more than 20 million people worldwide [1], with symptoms usually emerging in late adolescence or early adulthood [2]. Despite its global impact, the etiology of schizophrenia remains elusive, although the significant involvement of neuroinflammation in its diverse pathophysiological domains has been widely recognized [3]. In this context, drug-naïve first-episode psychotic (FEP) patients exhibit inflammatory abnormalities independent of medication use [4]. Furthermore, impairments in the glutathione (GSH) system compromise N-methyl-D-aspartate (NMDA) receptor activity, suggesting that oxidative damage is also a relevant mechanism contributing to cognitive impairment [5]. There is increasing evidence indicating an elevated risk of schizophrenia-related abnormalities resulting from neurodevelopmental defects caused by prenatal exposure to infections [6]. These findings propose the presence of various abnormalities with closely interconnected mechanisms within the brains of these patients. Thus, environmental risk factors can impact neuroproliferation and synaptic pruning, which are essential for healthy brain development during early prenatal periods and adolescence, respectively [7]. Consequently, these stages emerge as critical periods for the occurrence of high-risk abnormalities associated with subsequent neuropsychiatric conditions. Therefore, mitigating the impact of different environmental insults that may contribute to pathological neurodevelopmental imbalances could delay or prevent the onset of schizophrenia. Given the influential roles of adolescence and prenatal periods, both periods represent critical time windows for schizophrenia prevention [8].

Significant efforts have been made to develop effective strategies for predicting and preventing the transition to psychosis in high-risk patients [9]. However, current findings are still far from reliable. The ideal strategy would involve identifying patients at high risk for psychosis and implementing a treatment that can prevent their pathological transition without side effects. Accordingly, N-acetylcysteine (NAC) has garnered significant attention due to its remarkable ability to modulate impaired molecular systems in various psychiatric disorders, including schizophrenia [10, 11]. NAC, an antioxidant agent, plays a primary role in promoting GSH synthesis, thereby helping to restore oxidative homeostasis and neurotransmitter signaling [12]. With its low toxicity and minimal side effects profile [13], NAC has emerged as a promising candidate for preventive strategies in schizophrenia.

Previous preclinical studies have demonstrated some benefits of NAC treatment during adolescence as a preventive strategy [14, 15]. However, in clinical trials, NAC has only been evaluated as an adjuvant to antipsychotics [10], mainly due to the challenges associated with the early identification of high-risk patients. This suggests that earlier administration would be more appropriate. Notably, one study has already demonstrated the efficacy of a single injection of NAC following prenatal inflammation in three schizophrenia-like rat models [16]. Nonetheless, this approach requires almost immediate and invasive intervention, posing challenges for translation into the clinical setting. Therefore, there is a need for more translational strategies to evaluate the preventive potential of NAC during the prenatal period.

In this study, our initial aim was to assess the potential preventive effects of NAC administration during peri-adolescence in a maternal immune stimulation (MIS) rat model of schizophrenia. Building upon the promising findings, we further investigated NAC treatment during pregnancy in two different time windows: starting from the immune challenge or throughout the entire pregnancy. Notably, this study represents the first attempt to tackle this topic comprehensively using structural magnetic resonance imaging (MRI), diffusion tensor imaging (DTI), and MR spectroscopy (MRS), enabling us to evaluate in vivo structural and metabolic changes after NAC treatment. Additionally, we examined alterations in behavior and measured inflammation/oxidative stress (IOS) biomarkers, providing a broad perspective of the various levels affected in this animal model.

## Materials And Methods

### Animals

A total of 156 male Wistar rats were housed in a temperature- and humidity-controlled vivarium, following a 12 h dark/light cycle, and provided *ad libitum* access to standard rodent chow and tap water. Three separate batches of randomized animals were used for the different studies conducted. In the study performed during the periadolescent period, 84 rats were included, 35 animals from 17 litters for behavior (15 NAC, 20 Saline) and 49 animals for imaging (26 NAC, 23 Saline). In the study performed during the gestational period, 72 rats from 46 litters were included (48 NAC, 24 Saline). The sample size was estimated on previous studies [17–19]. All experimental animal procedures were conducted according to European Communities Council Directive 2010/63/EU and ARRIVE guidelines [20] and approved by the Ethics Committee for Animal Experimentation of Hospital Gregorio Marañón.

### Drug treatment and design of the study

At gestational day (GD) 15, pregnant Wistar rats were intravenously injected with Saline or PolyI:C (4 mg/kg, Sigma-Aldrich, batch 096M4023V) under sevoflurane anesthesia (3% induction, 1.5% maintenance in 100% O_2_). At postnatal day (PND) 21, animals were weaned, and only 2–3 males per litter were randomly selected and housed together (2–4 animals/cage).

NAC treatment during periadolescence: NAC (150 mg/kg, Sigma-Aldrich) or saline serum (VH) was administered via intraperitoneal injection between PND35–49. The study included four groups (12–14 animals/group) based on the experimental factors: phenotype (MIS, Saline) and treatment (NAC, VH).

**Fig. 1:**
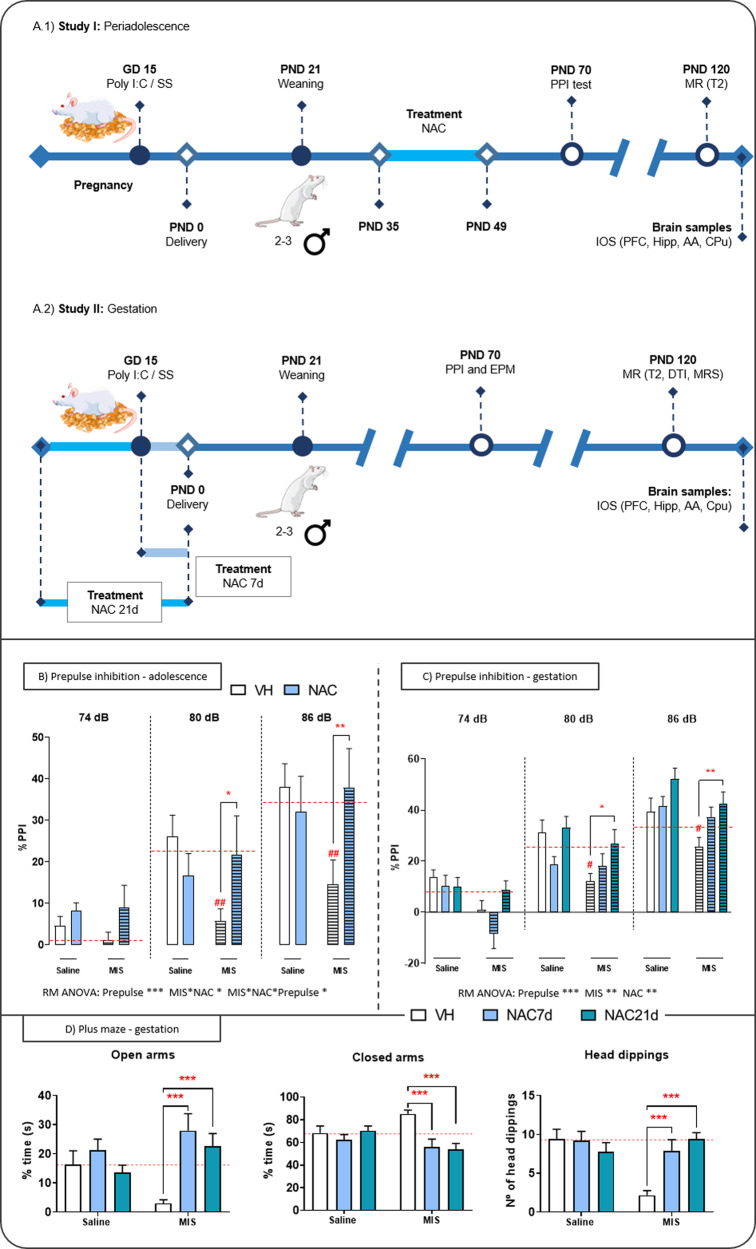
Study design and behavioral tests in periadolescence and gestation. **A** Scheme with the different experimental procedures for the periadolescence **(A.1)** and gestation **(A.2)** studies. **B** Prepulse inhibition (PPI) test in the periadolescence study. Each column represents mean ± SEM of the PPI (%) for each prepulse (PP) intensity (74, 80, and 86 dB) of 7–13 animals [Saline VH, *N* = 9; Saline NAC, *N* = 8; MIS VH, *N* = 11; MIS NAC, *N* = 7]. RM-ANOVA followed by Bonferroni post hoc test (*P < 0.05 vs. MIS VH; # *P* < 0.05 vs. Saline VH). **C** PPI test in the gestation study. Each column represents mean ± SEM of the PPI (%) for each prepulse PP intensity of 7–12 animals [Saline VH, *N* = 7; Saline NAC7d, *N* = 10; Saline NAC21d, *N* = 12; MIS VH, *N* = 7; MIS NAC7d, *N* = 10; MIS NAC21d, *N* = 12]. RM-ANOVA followed by Bonferroni post hoc test (*p < 0.05, **p < 0.01 vs. MIS VH; # *p* < 0.05 vs. Saline VH). **D** Elevated plus maze (EPM) test in gestation. Each column represents mean ± SEM of the time in closed (left) and open (middle) arms, together with the number of head dippings (right) [Saline VH, *N* = 8; Saline NAC7d, *N* = 12; Saline NAC21d, *N* = 12; MIS VH, *N* = 12; MIS NAC7d, *N* = 12; MIS NAC21d, *N* = 12]. Two-way ANOVA followed by Bonferroni post hoc test (**p < 0.01, ***p < 0.001 vs. MIS VH; ### *p* < 0.001 vs. Saline VH). Abbreviations: AA amygdala, CPu caudate putamen, DTI diffusion tensor imaging, GD gestational day, Hipp hippocampus, MRI magnetic resonance imaging, MRS magnetic resonance spectroscopy, PFC prefrontal cortex, PND postnatal day.

NAC treatment during gestation: NAC (500 mg/kg, Sigma-Aldrich) was administered in fresh drinking water (VH) during two different time windows: 1) from the immune challenge (GD15) until delivery (NAC7d); and 2) throughout the entire gestation (NAC21d). The study comprised six groups based on the experimental factors: phenotype (MIS, Saline) and treatment (VH, NAC7d, NAC21d). Figure 1A illustrates the design of the study.

### Behavioral studies

Behavioral studies were conducted between PND70–80 (early adulthood). Sensorimotor gating was assessed in all animals, while anxiety was measured specifically in the gestational study.

#### Prepulse inhibition of the startle reflex

Rats were individually placed in a startle chamber (Cibertec, Spain) and subjected to a pseudorandomized combination of the following stimuli [18, 19]: 1) 10 min acclimatization (background noise, 70 dB); 2) 5 trials of startle stimuli (pulse, 120 dB); 3) 10 trials which pseudorandomly presented pulse (120 dB), prepulse (74, 80, or 86 dB) + pulse (120 dB), or no stimulus (background noise); and 4) 5 trials of pulse (120 dB). Pulse and prepulse duration were 40 ms, the interval between prepulse and pulse was 100 ms, and the inter-trial interval ranged from 10 to 20 s. The percentage of prepulse inhibition (PPI) for each prepulse intensity was calculated as follows: 100 - [(startle response to prepulse + pulse) /startle response to pulse) x 100)]. The startle chamber was cleaned between each animal.

#### Elevated plus maze test

At PND80, anxiety levels were assessed using the elevated plus maze (EPM) test. The maze (Cibertec, Spain) consisted of two open arms and two enclosed arms at right angles (arms: 50 cm long, 40 cm high, 10 cm wide) raised 50 cm above the floor. Animals were placed in the center of the EPM, facing an open arm, and allowed to explore for 5 min. All experiments were recorded. The maze was cleaned between each animal using a 0.1% acetic acid solution. The total time spent in the open and closed arms, and the number of head dippings, were quantified.

#### In vivo magnetic resonance studies

Animals were scanned during adulthood (PND 100–120) using a 7-Tesla Biospec 70/20 scanner (Bruker, Germany) under sevoflurane anesthesia (3% induction, 1.5% maintenance in 100% O_2_). Rat-specific volume and surface coils were used. All animals were scanned for structural changes in gray matter. Diffusion tensor imaging (DTI) and spectroscopy studies were only performed on the animals participating in the gestational study (Supplementary Fig. 1).

##### Structural MRI imaging

Acquisition and reconstruction protocols were published elsewhere [18, 19]. Briefly, T2-weigthed Spin Echo (SE) coronal images were acquired with TE of 33 ms, TR of 3.732 ms and 0.4 mm in thickness. The matrix size was 256×256 pixels, covering a FOV of 3.5×3.5 cm^2^. The artifacts caused by the surface coil were corrected. Seven ROIs (PFC, whole cortex, hippocampus, cerebellum, ventricles, corpus callosum, and whole brain) were manually segmented [21], by two blinded operators, with in-depth anatomical knowledge, to reduce operator variability. This manual approach was used due to its robustness, as brain atlas-based segmentations for rodents still have some deficiencies in automated segmentation [22].

##### DTI study

Structural changes in white matter were evaluated using DTI. SE-EPI sequences were acquired, with TR of 2000 ms, TE of 26.55 ms, three repetitions, diffusion gradients applied in 27 directions (b value = 1000 s/mm^2^), 35 slices, 0.4 mm thickness, matrix size of 128 × 128 voxels, and FOV of 35 × 35 mm. Fractional anisotropy (FA) and mean diffusivity (MD) values were determined in the corpus callosum, PFC, and hippocampus.

##### MR spectroscopy

^1^H-MRS images were acquired using a PRESS mono-voxel sequence in the PFC and hippocampus, with the following parameters: TR of 2500 ms, TE of 16.5 ms, 2,048 acquisition points, 128 averages, bandwidth of 3301.06 Hz, and voxel size of 1.5 mm^3^, adjusted to the anatomical structure. An unsuppressed water signal was used as an internal reference for quantification. In vivo ^1^H-MRS spectra were analyzed using LCModel (Version 6.2-0U, S.W.Provencher) [23], considering only metabolites with a Cramer-Rao Lower Bound (CRLB) of less than 20%. Additionally, metabolites with overlapping resonances were presented as the combined sum of both species.

#### Postmortem IOS assessment

Biochemical determinations were performed on frozen tissue samples (−80 °C) obtained from the PFC, hippocampus, caudate-putamen, and amygdala.

##### Western blot

The inflammatory and oxidonitrosative inducible enzymes iNOS and COX-2, the cytoplasmic sequestrator of Nrf2 (KEAP1), and the Nrf2-targeting antioxidative genes HO1 and NQO1 were measured in cytosolic extracts, as previously reported [17, 19, 24]. Tissue was solubilized in a 50-mM Tris-hydrochloride buffer (pH 7.7) containing protease and phosphatase inhibitor cocktails (P2714, P5726; Sigma, Spain). Protein levels were assessed using the Bradford method. Expression levels of iNOS, COX-2, KEAP1, HO1, and NQO1 were determined using 15 µg of cytosolic extracts. Proteins were loaded onto an electrophoresis gel and subsequently blotted onto a membrane with a semi-dry transfer system. Blots were blocked with 5% BSA (Sigma, Spain) and incubated overnight at 4 °C with the following antibodies: rabbit anti-iNOS (sc-650, 1:750 BSA 2%; SCBT, USA), goat anti-COX2 (sc-1747, 1:750 BSA 2.5%; SCBT, USA), mouse anti-KEAP1 (MAB3024, 1:1000; R&D, USA), rabbit anti-HO1 (ab68477, 1:1000; Abcam, UK), goat anti-NQO1 (sc16464, 1:750 BSA 1%, SCBT, USA), and mouse anti-β-actin (A5441, 1:10000; Sigma, Spain). These primary antibodies were detected using horseradish peroxidase-linked secondary antibodies. Antibody binding was detected using an Odyssey Fc System (LI-COR®, Germany). All blots were performed at least three times in separate assays. Digital images of the Western Blot were analyzed using densitometry (ImageJ, NIH, USA). Values were normalized to the loading control (β-actin) and expressed as a percentage of variation compared to the control group.

##### Lipid peroxidation

The Thiobarbituric Acid Test for MDA was performed as described by Das and Ratty [25].

##### NRF2 activity

A commercial ELISA-based kit (600590, Cayman Chemical, Estonia) was used on nuclear extracts of tissue samples, following the manufacturer’s instructions.

##### Antioxidant enzyme activity [18]

Tissue samples were sonicated in 400 µL of PBS (pH = 7) containing a protease inhibitor cocktail (Complete©; Roche). Homogenates were centrifuged at 10,000 G for 15 min at 4 °C. Supernatants were used for determinations of superoxide dismutase (SOD, K028-H1, Arbor Assay, USA), catalase (CAT, K033-H1, Arbor Assay, USA), glutathione peroxidase (GPx, 703102, Cayman Chemical, Estonia), and glutathione (GSH, K006-H1, Arbor Assay, USA). Results were expressed as U/mg protein for the enzyme activities and in µM/µg protein for GSH levels.

### Statistical analyses

Normality and homoscedasticity were assessed by Shapiro–Wilk’s and Levene’s tests, respectively. Normal and homoscedastic variables were analyzed using a two-way ANOVA followed by a Bonferroni post hoc test. For the PPI results, a repeated-measures (RM) ANOVA was employed, considering phenotype, treatment, and prepulse intensity as factors. Non-normal and non-homoscedastic variables were analyzed using the Kruskal–Wallis (KW) test and Dunn’s post hoc test. A *p* value < 0.05 was considered statistically significant for all analyses. Statistical analyses were performed using SPSS (IBM SPSS Statistics 20, Spain), and graphic representations were generated using GraphPad Prism (v8, GraphPad Software, San Diego, CA, USA). Data are presented as mean ± SEM.

## RESULTS

Tables 1 and 2 provide a comprehensive overview of the results from the statistical analyses investigating the effects of NAC during periadolescence and pregnancy, respectively, for each of the conducted experiments.

**Table 1:** Statistical data of NAC-related effects during periadolescence on brain volumetry (A) and the expression of inflammatory and oxidative (IOS) markers (B).

ROIs	MIS		Treatment (NAC)		Interaction		KW
**A. Brain volumetry**							
Brain	F_(1,46)_ = 1.314	*p*=0.257	F_(1,46)_ = 1.378	*p*=0.246	F_(1,46)_ = 6.851	*p*=0.066	–
PFC	F_(1,46)_ = 0.444	*p*=0.510	F_(1,46)_ = 0.323	*p*=0.574	F_(1,46)_ = 3.019	*p*=0.089	–
Cx	–	–	–	–	–	–	0.243
Hipp	F_(1,46)_ = 7.642	*p*=0.008**	F_(1,46)_ = 5.289	*p*=0.026*	F_(1,46)_ = 0.943	*p*=0.334	–
V	F_(1,46)_ = 11.51	*p*=0.001**	F_(1,46)_ = 0.353	*p*=0.554	F_(1,46)_ < 0.001	*p*=0.977	–
CC	F_(1,46)_ = 149.5	*p*<0.001***	F_(1,46)_ = 0.353	*p*=0.555	F_(1,46)_ = 2.276	*p*=0.138	–
Cb	F_(1,46)_ = 1.292	*p*=0.262	F_(1,46)_ = 1.292	*p*=0.262	F_(1,46)_ = 3.624	*p*=0.063	–

Markers	MIS		Treatment (NAC)		Interaction		KW
**B. IOS markers**							
iNOS							
PFC	F_(1,28)_ = 20.240	*p*<0.001***	F_(1,28)_ = 10.010	*p*=0.004**	F_(1,28)_ = 6.851	*p*=0.014*	–
Hipp	F_(1,27)_ = 0.109	*p*=0.744	F_(1,27)_ = 1.291	*p*=0.266	F_(1,27)_ = 3.989	*p*=0.056	–
AA	F_(1,28)_ = 36.240	*p*<0.001***	F_(1,28)_ = <0.001	*p*=0.999	F_(1,28)_ = 2.556	*p*=0.121	–
Cpu	F_(1,28)_ = 5.406	*p*=0.027*	F_(1,28)_ = 3.241	*p*=0.083	F_(1,28)_ = 3.979	*p*=0.056	–
COX2							
PFC	F_(1,27)_ = 0.244	*p*=0.626	F_(1,27)_ = 0.818	*p*=0.374	F_(1,27)_ = 0.241	*p*=0.627	–
Hipp	F_(1,28)_ = 2.037	*p*=0.164	F_(1,28)_ = 5.021	*p*=0.033*	F_(1,28)_ = 0.006	*p*=0.939	–
AA	F_(1,28)_ = 15.080	*p*<0.001***	F_(1,28)_ = 5.457	*p*=0.027*	F_(1,28)_ = <0.001	*p*=0.987	–
Cpu	F_(1,28)_ = 0.148	*p*=0.704	F_(1,28)_ = 3.569	*p*=0.069	F_(1,28)_ = 0.900	*p*=0.351	–
MDA							
PFC	F_(1,28)_ = 11.940	*p*=0.002**	F_(1,28)_ = 0.199	*p*=0.659	F_(1,28)_ = 0.240	*p*=0.628	–
Hipp	F_(1,28)_ = 0.410	*p*=0.527	F_(1,28)_ = 0.209	*p*=0.651	F_(1,28)_ = 0.371	*p*=0.548	–
AA	F_(1,28)_ = 0.023	*p*=0.880	F_(1,28)_ = 0.132	*p*=0.719	F_(1,28)_ = 0.226	*p*=0.638	–
Cpu	–	–	–	–	–	–	*p*=0.398
KEAP1							
PFC	F_(1,27)_ = 0.455	*p*=0.506	F_(1,27)_ = 20.190	*p*<0.001***	F_(1,27)_ = 14.300	*p*<0.001***	–
Hipp	F_(1,28)_ = 3.496	*p*=0.072	F_(1,28)_ = 9.354	*p*=0.005**	F_(1,28)_ = 0.043	*p*=0.837	–
AA	F_(1,28)_ = 10.640	*p*=0.003**	F_(1,28)_ = 3.304	*p*=0.080	F_(1,28)_ = 1.731	*p*=0.199	–
Cpu	F_(1,27)_ = 0.927	*p*=0.344	F_(1,27)_ = 0.159	*p*=0.693	F_(1,27)_ = 1.047	*p*=0.315	–
HO1							
PFC	F_(1,28)_ = 0.039	*p*=0.845	F_(1,28)_ = 3.025	*p*=0.093	F_(1,28)_ = 1.406	*p*=0.246	–
Hipp	–	–	–	–	–	–	*p*=0.155
AA	F_(1,28)_ = 22.980	*p*<0.001***	F_(1,28)_ = 20.720	*p*<0.001***	F_(1,28)_ = 12.200	*p*=0.002**	–
Cpu	F_(1,28)_ = 0.102	*p*=0.752	F_(1,28)_ = 5.217	*p*=0.030*	F_(1,28)_ = 0.195	*p*=0.662	–
NQO1							
PFC	F_(1,28)_ = 0.317	*p*=0.578	F_(1,28)_ = 2.422	*p*=0.131	F_(1,28)_ = 4.324	*p*=0.047*	–
Hipp	F_(1,28)_ = 0.727	*p*=0.401	F_(1,28)_ = 1.734	*p*=0.199	F_(1,28)_ = 0.013	*p*=0.911	–
AA	F_(1,28)_ = 0.046	*p*=0.832	F_(1,28)_ = 4.853	*p*=0.036*	F_(1,28)_ = 5.084	*p*=0.032*	–
Cpu	–	–	–	–	–	–	*p*=0.927
NRF2							
PFC	F_(1,27)_ = 3.925	*p*=0.058	F_(1,27)_ = 0.032	*p*=0.859	F_(1,27)_ = 1.840	*p*=0.186	–
Hipp	F_(1,28)_ = 2.437	*p*=0.130	F_(1,28)_ = <0.001	*p*=0.978	F_(1,28)_ = 0.800	*p*=0.380	–
AA	F_(1,28)_ = 0.500	*p*=0.485	F_(1,28)_ = 4.784	*p*=0.037*	F_(1,28)_ = 0.134	*p*=0.037*	–
Cpu	–	–	–	–	–	–	*p*=0.888
SOD							
PFC	F_(1,28)_ = 1.608	*p*=0.215	F_(1,28)_ = 0.370	*p*=0.547	F_(1,28)_ = 1.331	*p*=0.258	–
Hipp	F_(1,28)_ = 0.053	*p*=0.819	F_(1,28)_ = 0.001	*p*=0.975	F_(1,28)_ = 1.315	*p*=0.261	–
AA	F_(1,28)_ = 0.780	*p*=0.384	F_(1,28)_ = 4.608	*p*=0.041*	F_(1,28)_ = 2.750	*p*=0.120	–
Cpu	F_(1,28)_ = 0.191	*p*=0.665	F_(1,28)_ = 1.379	*p*=0.250	F_(1,28)_ = 1.379	*p*=0.250	–
CAT							
PFC	F_(1,28)_ = 10.930	*p*=0.003**	F_(1,28)_ = 0.010	*p*=0.920	F_(1,28)_ = 0.976	*p*=0.331	–
Hipp	F_(1,28)_ = 3.171	*p*=0.086	F_(1,28)_ = 1.225	*p*=0.278	F_(1,28)_ = 0.049	*p*=0.826	–
AA	F_(1,28)_ = <0.001	*p*=0.999	F_(1,28)_ = 0.278	*p*=0.602	F_(1,28)_ = 3.978	*p*=0.056	–
Cpu	F_(1,28)_ = 3.741	*p*=0.063	F_(1,28)_ = 8.209	*p*=0.008**	F_(1,28)_ = 1.254	*p*=0.272	–
GPx							
PFC	–	–	–	–	–	–	*p*=0.110
Hipp	F_(1,28)_ = 0.281	*p*=0.600	F_(1,28)_ = 0.018	*p*=0.895	F_(1,28)_ = 0.021	*p*=0.886	–
AA	F_(1,28)_ = 0.003	*p*=0.957	F_(1,28)_ = 1.690	*p*=0.204	F_(1,28)_ = 1.469	*p*=0.236	–
Cpu	F_(1,28)_ = 6.042	*p*=0.020*	F_(1,28)_ = 1.681	*p*=0.205	F_(1,28)_ = 1.042	*p*=0.316	–
GSH_total_							
PFC	F_(1,28)_ = 8.428	*p*=0.007**	F_(1,28)_ = 0.538	*p*=0.469	F_(1,28)_ = 0.260	*p*=0.614	–
Hipp	F_(1,28)_ = <0.001	*p*=0.990	F_(1,28)_ = 0.585	*p*=0.585	F_(1,28)_ = 0.058	*p*=0.811	–
AA	F_(1,28)_ = 1.530	*p*=0.226	F_(1,28)_ = 0.451	*p*=0.508	F_(1,28)_ = 4.570	*p*=0.041*	–
Cpu	F_(1,28)_ = 4.380	*p*=0.045*	F_(1,28)_ = 1.629	*p*=0.212	F_(1,28)_ = 2.311	*p*=0.140	–
GSH_libre_							
PFC	F_(1,28)_ = 7.682	*p*=0.009**	F_(1,28)_ = 0.552	*p*=0.464	F_(1,28)_ = 0.067	*p*=0.798	–
Hipp	F_(1,28)_ = 0.281	*p*=0.600	F_(1,28)_ = 0.018	*p*=0.895	F_(1,28)_ = 0.021	*p*=0.886	–
AA	F_(1,28)_ = 1.929	*p*=0.176	F_(1,28)_ = 0.073	*p*=0.788	F_(1,28)_ = 4.105	*p*=0.052	–
Cpu	F_(1,28)_ = 3.883	*p*=0.059	F_(1,28)_ = 1.478	*p*=0.234	F_(1,28)_ = 2.081	*p*=0.160	–
GSSG							
PFC	F_(1,28)_ = 5.999	*p*=0.021*	F_(1,28)_ = 0.004	*p*=0.947	F_(1,28)_ = 1.278	*p*=0.268	–
Hipp	F_(1,28)_ = 0.037	*p*=0.845	F_(1,28)_ = 1.196	*p*=0.283	F_(1,28)_ = 0.014	*p*=0.905	–
AA	F_(1,28)_ = 0.313	*p*=0.580	F_(1,28)_ = 2.174	*p*=0.151	F_(1,28)_ = 3.515	*p*=0.071	–
Cpu	F_(1,28)_ = 4.926	*p*=0.035*	F_(1,28)_ = 1.791	*p*=0.192	F_(1,28)_ = 2.558	*p*=0.121	–

Each column represents the ANOVA F-test or Kruskal-Wallis test for MIS, NAC treatment, and its interaction for the studied areas. F: ANOVA F-test, KW: Kruskal-Wallis test (*p < 0.05, **p < 0.01, ***p < 0.001).

**Table 2:** Statistical data of NAC-related effects during pregnancy on anxiety (A), brain volumetry (B), brain DTI (C), brain MRS (D) and the expression of inflammatory and oxidative (IOS) markers (E).

ROIs	MIS		NAC 7d		Interaction		KW	MIS		NAC21		Interaction		KW
**A. Elevated plus maze (EPM)**														
Open arms	F_(1,40)_ = 0.612	*p*=0.438	F_(1,40)_ = 12.270	*p*=0.001**	F_(1,40)_ = 5.435	*p*=0.025*	–	F_(1,40)_ = 0.612	*p*=0.438	F_(1,40)_ = 12.270	*p=*0.001**	F_(1,40)_ = 5.435	*p*=0.025*	–
Closed arms	F_(1,40)_ = 0.841	*p*=0.365	F_(1,40)_ = 9.906	*p*=0.003**	F_(1,40)_ = 4.234	*p*=0.044*	–	F_(1,40)_ = 0.841	*p*=0.365	F_(1,40)_ = 9.906	*p*=0.003**	F_(1,40)_ = 4.234	*p*=0.044*	–
Head dippings	F_(1,40)_ = 12.380	*p*=0.001**	F_(1,40)_ = 5.053	*p*=0.030*	F_(1,40)_ = 5.854	*p*=0.020*	–	F_(1,40)_ = 12.380	*p*=0.001**	F_(1,40)_ = 5.053	*p*=0.030*	F_(1,40)_ = 5.854	*p*=0.020*	–
**B. Brain volumetry**														
Brain	F_(1,33)_ = 18.620	*p*<0.001***	F_(1,33)_ = 1.077	*p*=0.307	F_(1,33)_ = 0.171	*p*=0.682	–	F_(1,33)_ = 1.662	*p*=0.206	F_(1,33)_ = 0.842	*p*=0.365	F_(1,33)_ = 9.351	*p*=0.004**	–
PFC	F_(1,33)_ = 10.780	*p*=0.002**	F_(1,33)_ = 6.336	*p*=0.017*	F_(1,33)_ = 0.002	*p*=0.968	–	F_(1,33)_ = 3.177	*p*=0.084	F_(1,33)_ = 0.600	*p*=0.447	F_(1,33)_ = 2.898	*p*=0.099	–
Cx	F_(1,33)_ = 3.080	*p*=0.089	F_(1,33)_ = 10.340	*p*=0.003**	F_(1,33)_ = 4.063	*p*=0.052	–	F_(1,33)_ = 0.985	*p*=0.328	F_(1,33)_ = 1.245	*p*=0.273	F_(1,33)_ = 0.547	*p*=0.465	–
HP	F_(1,33)_ = 9.856	*p*=0.004**	F_(1,33)_ = 9.830	*p*=0.004**	F_(1,33)_ = 2.513	*p*=0.122	–	F_(1,33)_ = 6.560	*p*=0.015*	F_(1,33)_ = 6.319	*p*=0.017*	F_(1,33)_ = 5.512	*p*=0.025*	–
V	F_(1,32)_ = 4.498	*p*=0.042*	F_(1,32)_ = 1.409	*p*=0.244	F_(1,32)_ = 6.394	*p*=0.017*	–	F_(1,32)_ = 3.980	*p*=0.055	F_(1,32)_ = 0.392	*p*=0.536	F_(1,32)_ = 2.325	*p*=0.137	–
CC	F_(1,32)_ = 4.987	*p*=0.033*	F_(1,32)_ = 35.210	*p*<0.001***	F_(1,32)_ = 5.100	*p*=0.031*	–	F_(1,32)_ = 0.056	*p*=0.814	F_(1,32)_ = 32.030	*p*<0.001***	F_(1,32)_ = 22.750	*p*<0.001***	–
Cb	F_(1,33)_ = 15.240	*p*<0.001***	F_(1,33)_ = 5.875	*p*=0.021*	F_(1,33)_ = 0.312	*p*=0.576	–	F_(1,33)_ = 2.393	*p=*0.436	F_(1,33)_ = 0.622	*p*=0.436	F_(1,33)_ = 9.220	*p*=0.005**	–
**C. Diffusion tensor imaging (DTI)**														
FA														
CC	F_(1,33)_ = 1.343	*p*=0.255	F_(1,33)_ = 2.966	*p*=0.094	F_(1,33)_ = 26.040	*p*<0.001***	–	F_(1,33)_ = 10.990	*p*=0.002**	F_(1,33)_ = 16.400	*p*<0.001***	F_(1,33)_ = 6.527	*p*=0.015*	–
PFC	–	–	–	–	–	–	*p*=0.919	–	–	–	–	–	–	*p*=0.919
HP	F_(1,31)_ = 0.195	*p*=0.662	F_(1,31)_ = 2.452	*p*=0.127	F_(1,31)_ = 0.272	*p*=0.606	–	F_(1,31)_ = 0.195	*p*=0.662	F_(1,31)_ = 2.452	*p*=0.127	F_(1,31)_ = 0.272	*p*=0.606	–
MD														
CC	–	–	–	–	–	–	*p*=0.002**	–	–	–	–	–	–	*p*=0.002**
PFC	–	–	–	–	–	–	*p*=0.001**	–	–	–	–	–	–	*p*=0.001**
HP	–	–	–	–	–	–	*p*<0.001***	–	–	–	–	–	–	*p*<0.001***
**D. Magnetic resonance spectroscopy (MRS)**														
FA														
GPC+PCh	F_(1,26)_ = 0.001	*p*=0.968	F_(1,26)_ = 0.652	*p*=0.427	F_(1,26)_ = 0.676	*p*=0.418	–	F_(1,28)_ = 2.220	*p*=0.147	F_(1,28)_ = 1.317	*p*=0.261	F_(1,28)_ = 1.178	*p*=0.287	–
NAA+NAAG	F_(1,31)_ = 0.105	*p*=0.747	F_(1,31)_ = 0.024	*p*=0.878	F_(1,31)_ = 0.472	*p*=0.497	–	F_(1,31)_ = 2.911	*p*=0.098	F_(1,31)_ = <0.001	*p*=0.983	F_(1,31)_ = 0.396	*p*=0.534	–
Glu+Gln	F_(1,31)_ = 0.139	*p*=0.712	F_(1,31)_ = 1.114	*p*=0.299	F_(1,31)_ = 0.041	*p*=0.840	–	–	–	–	–	–	–	*p*=0.270
MM09+Lip09	F_(1,31)_ = 0.622	*p*=0.436	F_(1,31)_ = 2.037	*p*=0.163	F_(1,31)_ = 0.938	*p*=0.340	–	F_(1,30)_ = 4.802	*p*=0.036*	F_(1,30)_ = 11.340	*p*=0.002**	F_(1,30)_ = 1.635	*p*=0.211	–
MM20+Lip20	F_(1,30)_ = 2.056	*p*=0.162	F_(1,30)_ = 0.443	*p*=0.511	F_(1,30)_ = 0.032	*p*=0.860	–	F_(1,25)_ = 5.405	*p*=0.028*	F_(1,25)_ = 8.929	*p*=0.006**	F_(1,25)_ = 0.189	*p*=0.669	–
MD														
GPC+PCh	F_(1,26)_ = 0.500	*p*=0.487	F_(1,26)_ = 4.538	*p*=0.043*	F_(1,26)_ = 0.111	*p*=0.742	–	F_(1,28)_ = 6.303	*p*=0.018*	F_(1,28)_ = 7.612	*p*=0.010*	F_(1,28)_ = 0.015	*p*=0.903	–
NAA+NAAG	F_(1,31)_ = 0.118	*p*=0.734	F_(1,31)_ = 2.856	*p*=0.101	F_(1,31)_ = 0.001	*p*=0.972	–	F_(1,31)_ = 0.779	*p*=0.384	F_(1,31)_ = 0.691	*p*=0.412	F_(1,31)_ = 0.800	*p*=0.379	–
Glu+Gln	F_(1,31)_ = 0.702	*p*=0.408	F_(1,31)_ = 0.097	*p*=0.757	F_(1,31)_ = 0.460	*p*=0.502	–	–	–	–	–	–	–	*p*=0.270
MM09+Lip09	F_(1,31)_ = 0.451	*p*=0.507	F_(1,31)_ = 8.367	*p*=0.007**	F_(1,31)_ = 0.183	*p*=0.671	–	F_(1,30)_ = 0.011	*p*=0.918	F_(1,30)_ = 3.438	*p*=0.074	F_(1,30)_ = 0.778	*p*=0.385	–
MM20+Lip20	F_(1,29)_ = 0.244	*p*=0.625	F_(1,29)_ = 4.716	*p*=0.038*	F_(1,29)_ = 0.829	*p*=0.0370	–	F_(1,25)_ = 0.549	*p*=0.465	F_(1,25)_ = 6.019	*p*=0.021*	F_(1,25)_ = 0.795	*p*=0.381	–
**E. IOS markers**														
iNOS														
PFC	F_(1,27)_ = 7.453	*p=*0.011*	F_(1,27)_ = 11.120	*p=*0.002**	F_(1,27)_ = 4.373	*p*=0.046*	–	F_(1,27)_ = 23.220	*p*<0.001***	F_(1,27)_ = 2.970	*p*<0.001***	F_(1,27)_ = 5.639	*p*=0.025*	–
HP	F_(1,27)_ = 0.918	*p=*0.346	F_(1,27)_ = 8.366	*p*=0.007**	F_(1,27)_ = 6.408	*p*=0.017*	–	F_(1,27)_ = 0.752	*p=*0.393	F_(1,27)_ = 4.470	*p*=0.044*	F_(1,27)_ = 6.369	*p*=0.018*	–
AA	F_(1,27)_ = 10.700	*p=*0.003**	F_(1,27)_ = 0.566	*p*=0.458	F_(1,27)_ = 2.813	*p*=0.105	–	F_(1,27)_ = 7.430	*p*=0.011*	F_(1,27)_ = <0.001	*p*=0.986	F_(1,27)_ = 4.905	*p*=0.035*	–
CPu	F_(1,27)_ = 0.833	*p=*0.369	F_(1,27)_ = 0.169	*p*=0.684	F_(1,27)_ = 0.006	*p=*0.937	–	F_(1,26)_ = 1.045	*p=*0.316	F_(1,26)_ = 0.366	*p*=0.550	F_(1,26)_ = 0.075	*p*=0.787	–
COX2														
PFC	F_(1,27)_ = 1.169	*p*=0.289	F_(1,27)_ = 1.901	*p=*0.179	F_(1,27)_ = 2.013	*p*=0.167	–	F_(1,27)_ = 1.543	*p*=0.225	F_(1,27)_ = 2.661	*p=*0.114	F_(1,27)_ = 2.086	*p*=0.160	–
HP	F_(1,27)_ = 13.800	*p*<0.001***	F_(1,27)_ = 0.690	*p*=0.413	F_(1,27)_ = 1.083	*p=*0.307	–	F_(1,27)_ = 6.916	*p=*0.014*	F_(1,27)_ = 0.086	*p*=0.772	F_(1,27)_ = 3.411	*p*=0.076	–
AA	F_(1,26)_ = 4.663	*p*=0.040*	F_(1,26)_ = 2.780	*p<*0.001***	F_(1,26)_ = 3.090	*p*=0.090	–	F_(1,27)_ = 5.586	*p=*0.026*	F_(1,27)_ = 7.221	*p*=0.012*	F_(1,27)_ = 1.293	*p*=0.266	–
CPu	F_(1,25)_ = 5.708	*p*=0.025*	F_(1,25)_ = 0.245	*p*=0.625	F_(1,25)_ = 0.662	*p*=0.423	–	F_(1,24)_ = 6.129	*p*=0.021*	F_(1,24)_ = 4.773	*p*=0.039*	F_(1,24)_ = 0.472	*p*=0.498	–
MDA														
PFC	F_(1,27)_ = 1.655	*p*=0.209	F_(1,27)_ = 4.152	*p=*0.051	F_(1,27)_ = 0.989	*p*=0.329	–	F_(1,27)_ = 3.594	*p*=0.069	F_(1,27)_ = 7.572	*p*=0.010*	F_(1,27)_ = 5.044	*p*=0.033*	–
HP	–	–	–	–	–	–	*p*=0.727	–	–	–	–	–	–	*p*=0.727
AA	–	–	–	–	–	–	*p*=0.385	–	–	–	–	–	–	*p*=0.385
CPu	F_(1,27)_ = 0.010	*p*=0.921	F_(1,27)_ = 0.716	*p*=0.405	F_(1,27)_ = 0.834	*p*=0.369	–	F_(1,27)_ = 6.675	*p*=0.015*	F_(1,27)_ = 1.788	*p*=0.192	F_(1,27)_ = 1.661	*p*=0.208	–
KEAP1														
PFC	F_(1,27)_ = 0.662	*p=*0.423	F_(1,27)_ = 2.190	*p=*0.150	F_(1,27)_ = 0.810	*p*=0.376	–	F_(1,27)_ = 0.044	*p*=0.835	F_(1,27)_ = 2.217	*p*=0.148	F_(1,27)_ = 1.276	*p*=0.269	–
HP	F_(1,27)_ = 2.370	*p*=0.135	F_(1,27)_ = 2.398	*p*=0.133	F_(1,27)_ = 0.130	*p*=0.722	–	F_(1,27)_ = 0.172	*p=*0.682	F_(1,27)_ = 2.194	*p*=0.150	F_(1,27)_ = 0.404	*p*=0.531	–
AA	F_(1,26)_ = 1.309	*p*=0.263	F_(1,26)_ = 0.405	*p*=0.530	F_(1,26)_ = 5.436	*p*=0.028*	–	F_(1,27)_ = 0.286	*p*=0.597	F_(1,27)_ = 0.225	*p*=0.639	F_(1,27)_ = 0.389	*p*=0.538	–
CPu	F_(1,25)_ = 6.858	*p=*0.015*	F_(1,25)_ = 7.368	*p=*0.012*	F_(1,25)_ = 1.789	*p*=0.193	–	F_(1,26)_ = 5.487	*p*=0.027*	F_(1,26)_ = 2.468	128	F_(1,26)_ = 1.619	*p*=0.214	–
HO1														
PFC	–	–	–	–	–	–	*p*=0.568	–	–	–	–	–	–	*p*=0.568
HP	–	–	–	–	–	–	*p*=0.332	–	–	–	–	–	–	*p*=0.332
AA	–	–	–	–	–	–	*p*=0.193	–	–	–	–	–	–	*p*=0.193
CPu	F_(1,27)_ = 5.681	*p*=0.024*	F_(1,27)_ = 1.742	*p*=0.198	F_(1,27)_ = 0.024	*p*=0.879	–	F_(1,26)_ = 0.002	*p=*0.962	F_(1,26)_ = 1.226	*p*=0.278	F_(1,26)_ = 6.535	*p*=0.017*	–
NQO1														
PFC							*p*=0.247							*p*=0.247
HP	F_(1,26)_ = 2.078	*p=*0.161	F_(1,26)_ = 1.611	*p*=0.215	F_(1,26)_ = 0.214	*p*=0.648	–	F_(1,27)_ = 0.415	*p*=0.525	F_(1,27)_ = 5.583	*p*=0.026*	F_(1,27)_ = 4.038	*p*=0.055	–
AA	F_(1,27)_ = 0.791	*p*=0.382	F_(1,27)_ = 3.780	*p=*0.062	F_(1,27)_ = 0.039	*p*=0.844	–	F_(1,27)_ = 0.056	*p=*0.814	F_(1,27)_ = 12.640	*p*=0.001**	F_(1,27)_ = 1.258	*p*=0.272	–
CPu	F_(1,27)_ = 0.212	*p*=0.649	F_(1,27)_ = 8.577	*p=*0.007**	F_(1,27)_ = 0.668	*p*=0.421	–	F_(1,26)_ = <0.001	*p=*0.979	F_(1,26)_ = 9.103	*p*=0.006**	F_(1,26)_ = 1.106	*p*=0.303	–
NRF2														
PFC	–	–	–	–	–	–	*p*=0.011*	–	–	–	–	–	–	*p*=0.011*
HP	–	–	–	–	–	–	*p*=0.154	–	–	–	–	–	–	*p*=0.154
AA	F_(1,26)_ = 6.484	*p=*0.017*	F_(1,26)_ = 0.309	*p=*0.583	F_(1,26)_ = 0.134	*p=*0.718	–	F_(1,27)_ = 4.026	*p*=0.055	F_(1,27)_ = 3.371	*p*=0.077	F_(1,27)_ = 0.893	*p=*0.353	–
CPu	F_(1,27)_ = 2.804	*p*=0.106	F_(1,27)_ = 2.565	*p=*0.121	F_(1,27)_ = 0.001	*p*=0.971	–	F_(1,27)_ = 0.004	*p*=0.950	F_(1,27)_ = 0.214	*p*=0.647	F_(1,27)_ = 3.699	*p*=0.065	–
SOD								–	–	–	–	–	–	
PFC	F_(1,27)_ = 0.495	*p*=0.488	F_(1,27)_ = 1.469	*p*=0.236	F_(1,27)_ = 0.031	*p*=0.862	–	F_(1,27)_ = 1.095	*p*=0.305	F_(1,27)_ = 2.094	*p=*0.159	F_(1,27)_ = 2.937	*p*=0.098	–
HP	F_(1,27)_ = 0.740	*p*=0.397	F_(1,27)_ = 0.219	*p*=0.643	F_(1,27)_ = 2.838	*p=*0.104	–	F_(1,27)_ = 0.077	*p*=0.783	F_(1,27)_ = 0.116	*p*=0.736	F_(1,27)_ = 1.451	*p*=0.239	–
AA	F_(1,26)_ = 0.030	*p=*0.863	F_(1,26)_ = 1.361	*p*=0.254	F_(1,26)_ = 0.008	*p*=0.929	–	F_(1,26)_ = 1.600	*p*=0.217	F_(1,26)_ = 4.234	*p*=0.049*	F_(1,26)_ = 1.074	*p*=0.310	–
CPu	F_(1,27)_ = 0.007	*p*=0.932	F_(1,27)_ = 1.441	*p*=0.240	F_(1,27)_ = 2.407	*p=*0.132	–	F_(1,27)_ = 0.001	*p*=0.968	F_(1,27)_ = 3.617	*p*=0.068	F_(1,27)_ = 2.458	*p*=0.129	–
CAT														
PFC	F_(1,26)_ = 0.531	*p*=0.473	F_(1,26)_ = 3.599	*p*=0.069	F_(1,26)_ = 0.287	*p*=0.596	–	F_(1,26)_ = 0.357	*p*=0.555	F_(1,26)_ = 0.864	*p*=0.361	F_(1,26)_ = 0.287	*p*=0.597	–
HP	F_(1,26)_ = 0.300	*p*=0.589	F_(1,26)_ = 11.840	*p*=0.02**	F_(1,26)_ = 0.561	*p*=0.460	–	F_(1,26)_ = 0.066	*p*=0.799	F_(1,26)_ = 31.500	*p*<0.001***	F_(1,26)_ = 3.507	*p*=0.072	–
AA	F_(1,26)_ = 0.606	*p=*0.443	F_(1,26)_ = 3.783	*p=*0.063	F_(1,26)_ = 0.335	*p*=0.567	–	F_(1,26)_ = 1.701	*p*=0.204	F_(1,26)_ = 6.670	*p*=0.016*	F_(1,26)_ = 0.007	*p*=0.933	–
CPu	F_(1,27)_ = 5.517	*p*=0.026*	F_(1,27)_ = 3.130	*p*=0.088	F_(1,27)_ = 3.742	*p*=0.064	–	F_(1,27)_ = 1.195	*p*=0.284	F_(1,27)_ = 9.128	*p=*0.005**	F_(1,27)_ = 0.405	*p*=0.530	–
GPx														
PFC	F_(1,27)_ = 3.188	*p*=0.085	F_(1,27)_ = 0.012	*p*=0.912	F_(1,27)_ = 0.034	*p*=0.855	–	F_(1,27)_ = 3.794	*p*=0.062	F_(1,27)_ = 0.127	*p*=0.725	F_(1,27)_ = 0.002	*p*=0.966	–
HP	F_(1,27)_ = 0.768	*p*=0.389	F_(1,27)_ = 0.254	*p*=0.618	F_(1,27)_ = 0.280	*p*=0.867	–	F_(1,27)_ = 0.114	*p*=0.738	F_(1,27)_ = 0.175	*p*=0.679	F_(1,27)_ = 0.692	*p*=0.413	–
AA	F_(1,27)_ = 0.141	*p=*0.710	F_(1,27)_ = <0.001	*p*=0.978	F_(1,27)_ = 2.002	*p*=0.169	–	F_(1,27)_ = 0.021	*p*=0.884	F_(1,27)_ = 0.905	*p*=0.350	F_(1,27)_ = 0.966	*p*=0.334	–
CPu	F_(1,27)_ = 0.021	*p=*0.885	F_(1,27)_ = 3.404	*p=*0.76	F_(1,27)_ = 1.779	*p=*0.193	–	F_(1,27)_ = 5.762	*p*=0.023*	F_(1,27)_ = 0.174	*p*=0.680	F_(1,27)_ = 0.168	*p*=0.685	–
Total GSH														
PFC	F_(1,27)_ = 0.007	*p*=0.933	F_(1,27)_ = 0.189	*p*=0.667	F_(1,27)_ = 0.013	*p*=0.910	–	F_(1,27)_ = 1.091	*p*=0.305	F_(1,27)_ = 0.119	*p*=0.733	F_(1,27)_ = 1.155	*p*=0.292	–
HP	–	–	–	–	–	–	*p*=0.698	–	–	–	–	–	–	*p*=0.698
AA	F_(1,27)_ = 0.694	*p*=0.412	F_(1,27)_ = 0.473	*p*=0.498	F_(1,27)_ = 3.241	*p*=0.083	–	F_(1,27)_ = 0.024	*p*=0.877	F_(1,27)_ = 0.283	*p*=0.599	F_(1,27)_ = 0.883	*p*=0.356	–
CPu	F_(1,27)_ = 0.051	*p*=0.824	F_(1,27)_ = 0.171	*p=*0.682	F_(1,27)_ = 0.140	*p*=0.711	–	F_(1,27)_ = 0.698	*p*=0.411	F_(1,27)_ = 1.774	*p*=0.194	F_(1,27)_ = 0.425	*p*=0.520	–
Free GSH														
PFC	F_(1,27)_ = 0.106	*p=*0.747	F_(1,27)_ = 1.206	*p*=0.282	F_(1,27)_ = <0.001	*p=*0.983	–	F_(1,27)_ = 1.455	*p=*0.238	F_(1,27)_ = 0.025	*p*=0.876	F_(1,27)_ = 0.733	*p*=0.399	–
HP	–	–	–	–	–	–	*p*=0.754	–	–	–	–	–	–	*p*=0.754
AA	F_(1,27)_ = 0.802	*p*=0.378	F_(1,27)_ = 0.002	*p*=0.964	F_(1,27)_ = 3.013	*p*=0.094	–	F_(1,27)_ = 0.011	*p*=0.918	F_(1,27)_ = 0.783	*p*=0.384	F_(1,27)_ = 0.673	*p*=0.419	–
CPu	F_(1,27)_ = 0.094	*p*=0.762	F_(1,27)_ = 0.137	*p*=0.714	F_(1,27)_ = 0.456	*p*=0.505	–	F_(1,27)_ = 1.167	*p=*0.289	F_(1,27)_ = 0.047	*p*=0.830	F_(1,27)_ = 0.382	*p=*0.542	–
GSSG														
PFC	F_(1,27)_ = <0.001	*p=*0.979	F_(1,27)_ = 0.015	*p*=0.903	F_(1,27)_ = 0.018	*p*=0.894	–	F_(1,27)_ = 0.731	*p*=0.400	F_(1,27)_ = 0.519	*p*=0.478	F_(1,27)_ = 0.932	*p*=0.343	–
HP	–	–	–	–	–	–	*p*=0.446	–	–	–	–	–	–	*p*=0.446
AA	F_(1,27)_ = 0.034	*p*=0.855	F_(1,27)_ = 7.118	*p*=0.013*	F_(1,27)_ = 4.446	*p*=0.044*	–	F_(1,27)_ = 0.025	*p*=0.874	F_(1,27)_ = 0.502	*p*=0.485	F_(1,27)_ = 2.423	*p*=0.131	–
CPu	F_(1,27)_ = 0.001	*p*= 0.970	F_(1,27)_ = 3.939	*p*=0.57	F_(1,27)_ = 0.077	*p*=0.783	–	F_(1,27)_ = 0.082	*p*=0.777	F_(1,27)_ = 11.160	*p*=0.002**	F_(1,27)_ = 0.429	*p*=0.518	–

Each column represents the ANOVA F-test or Kruskal-Wallis test for MIS, NAC treatment, and its interaction for the studied areas. F: ANOVA F-test, KW: Kruskal-Wallis test (*p < 0.05, **p < 0.01, ***p < 0.001).

### Behavioral studies

#### Prepulse inhibition of the startle reflex

The PPI assesses deficits in sensorimotor gating, a common symptom in schizophrenia, by evaluating the suppression of an acoustic startle reflex to an intense stimulus when pre-exposed to a weak stimulus. We confirmed the presence of these deficits in our model, as both the periadolescence and gestational MIS-VH groups exhibited a significant PPI reduction at 80 dB and 86 dB (vs. Saline-VH group). In the periadolescence study, the RM-ANOVA revealed a significant prepulse effect (*p* < 0.001), an interaction between MIS and treatment (*p* < 0.05), and a three-way interaction (prepulse, MIS, and NAC) (*p* < 0.05) (Fig. 1C). Post hoc tests indicated that NAC did not significantly alter PPI values in the VH animals, but it prevented the PPI reduction in the MIS-offspring (vs. MIS-VH group). In the gestational study, the RM-ANOVA revealed significant effects of MIS (*p* < 0.01), NAC treatment (*p* < 0.01), and prepulse (*p* < 0.001) (Fig. 1D). Post hoc tests showed that NAC7d did not significantly improve PPI in the MIS-offspring, whereas NAC21d fully prevented this deficit (vs. MIS-VH group).

#### Elevated plus maze

The EPM (Fig. 1E) was used to evaluate anxiety levels in this model. Consistent with expectations, the MIS-VH animals spent more time in the closed arms and less time in the open arms compared to the Saline-VH rats, indicating higher anxiety levels. Additionally, the MIS-VH group exhibited a significant reduction in the number of head-dippings (*p* < 0.001). Importantly, both the NAC7d and NAC21d treatments completely prevented these deficits in the MIS-offspring.

### MRI studies

In adulthood, T2-weighted images were acquired to evaluate changes in regional volumetry. Moreover, DTI and metabolite content data were obtained in the gestational approach.

#### Volumetry

**Fig. 2:**
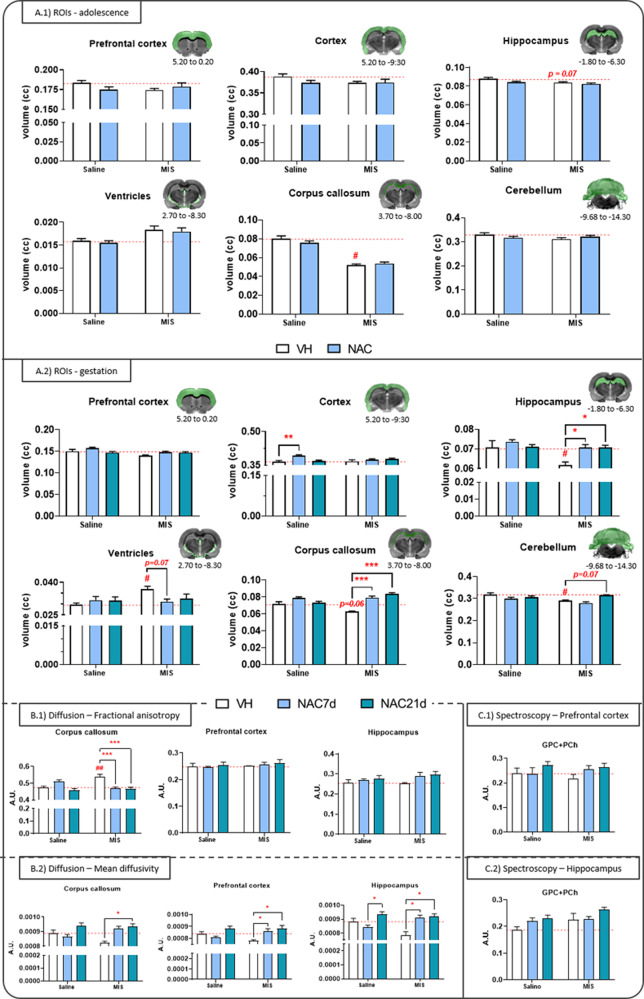
Volumetric and metabolic changes measured via MRI. **A** Regional volumetric changes in adulthood after NAC treatment during adolescence **(A.1))** [Saline VH, *N* = 11; Saline NAC, *N* = 12; MIS VH, *N* = 12; MIS NAC, *N* = 14] and in the offspring from dams that received NAC treatment during pregnancy **(A.2) [**Saline VH, *N* = 7; Saline NAC7d, *N* = 12; Saline NAC21d, *N* = 12; MIS VH, *N* = 6; MIS NAC7d, *N* = 12; MIS NAC21d, *N* = 12]. Each column represents mean ± SEM of the six brain areas volume (cc). Two-way ANOVA followed by Bonferroni post-hoc test (*p < 0.05, **p < 0.01, ***p < 0.001 vs. MIS VH; # *p* < 0.05 vs. Saline VH). **B** DTI results in the offspring from dams that received NAC treatment during pregnancy. Fraction anisotropy (FA) **(B.1)** and mean diffusivity (MD) **(B.2)** results in corpus callosum, prefrontal cortex, and hippocampus. Each column represents mean ± SEM of FA and MD (A.U.) [Saline VH, *N* = 7; Saline NAC7d, *N* = 12; Saline NAC21d, *N* = 12; MIS VH, *N* = 6; MIS NAC7d, *N* = 12; MIS NAC21d, *N* = 12]. Two-way ANOVA followed by Bonferroni post-hoc test (*p < 0.05, ***p < 0.001 vs. MIS VH; ## *p* < 0.01 vs. Saline VH. **C** MRS results in the offspring from dams that received NAC treatment during pregnancy in the prefrontal cortex. Each column represents mean ± SEM of the relative concentration of three brain metabolites with CRLB < 20% (glycerophosphocholine and phosphocholine, GPc + PCh; N-acetylaspartate and N-acetylaspartylglutamate, NAA + NAAG; glutamate and glutamine, Glu + Gln). [Saline VH, *N* = 7; Saline NAC7d, *N* = 12; Saline NAC21d, *N* = 12; MIS VH, *N* = 6; MIS NAC7d, *N* = 12; MIS NAC21d, *N* = 12]. Two-way ANOVA followed by Bonferroni post-hoc test.

Six brain regions were segmented on T2-weighted images and analyzed using a regions-of-interest (ROIs) approach. In the periadolescence study (Fig. 2A.1), the MIS-VH animals exhibited significant ventricular enlargement and reduced volume in the hippocampus and corpus callosum (vs. Saline-VH rats) (*p* < 0.01 in all cases), although none of these deficits were prevented by NAC. In the gestational study (Fig. 2A.2), the MIS-VH animals displayed reduced volume in the hippocampus, cerebellum, and nearly significant reduction in the corpus callosum (*p* = 0.06), along with ventricular enlargement (*p* < 0.05). In this case, both NAC7d and NAC21d prevented the hippocampal and corpus callosum deficits. Furthermore, we observed a trend towards the prevention of volumetric abnormalities in the ventricles (*p* = 0.07) and cerebellum (*p* = 0.07) by NAC7d and NAC21, respectively. Finally, the Saline-NAC7d offspring exhibited increased cortical volume (*p* < 0.01).

#### DTI

Differences in FA and MD were assessed through ROI analyses of the DTI images. The MIS-VH animals exhibited an increase in FA in the corpus callosum (*p* < 0.01, vs. Saline-VH), which was prevented by both NAC7d and NAC21d (*p* < 0.001) (Fig. 2B.1). No significant differences in FA were observed in other ROIs. Conversely, significant effects of NAC7d and NAC21d were observed (*p* < 0.05), restoring MD values in the prefrontal cortex (PFC) and hippocampus (Fig. 2B.2).

#### MRS

The uni-voxel spectroscopy data did not reveal statistically significant differences for any of the metabolites. However, a noticeable trend indicating a time-dependent effect of NAC was observed for choline in the PFC (Fig. 2C.1) and hippocampus (Fig.2C.2), as well as for N-acetyl aspartate in the PFC.

### IOS determinations

**Fig. 3:**
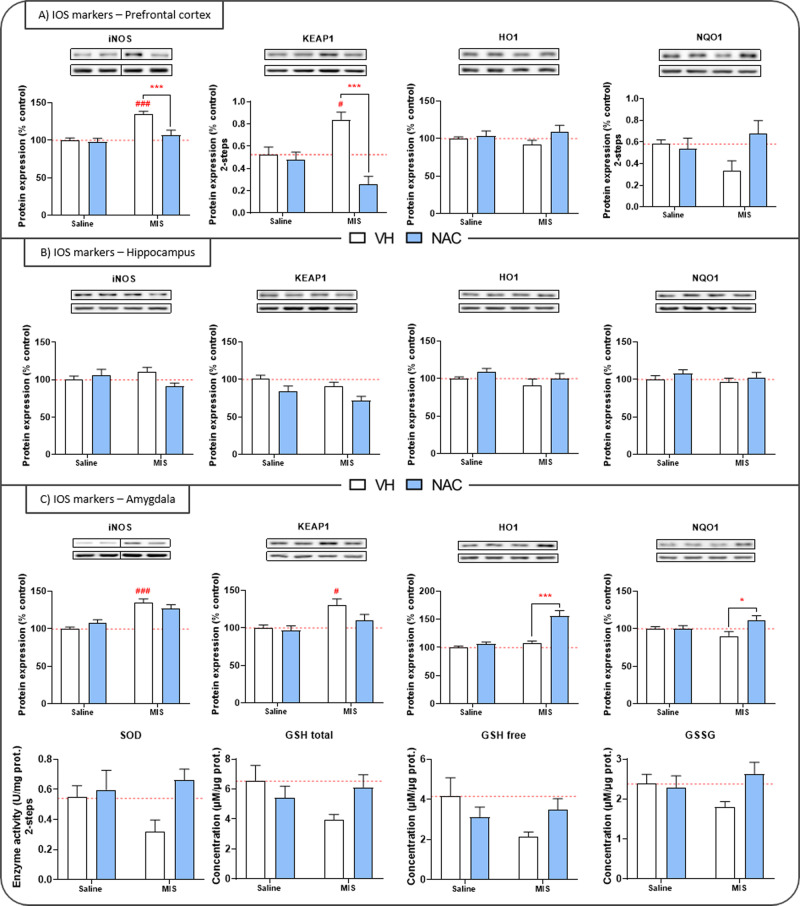
Inflammatory and oxidative stress (IOS) biomarkers in the periadolescence study. Inflammatory markers expression (iNOS, COX2), antioxidant enzyme activity (GPx, CAT, SOD), and oxidative stress markers (MDA, NRF2, KEAP1, HO1, NQO1, GSHf_ree_, GSH_total_, GSSG) in the prefrontal cortex (**A**), hippocampus (**B**), and amygdala (**C**). Representative bands of iNOS, COX2, KEAP1, HO1, and NQO1 (upper bands) and the loading control, β-actin (lower bands), are shown above their corresponding graph bars. Each column represents mean ± SEM of the protein expression (% control), enzymatic activity (U/mg protein), or concentration (µM/µg protein) of eight animals per group. Two-way ANOVA followed by Bonferroni post-hoc test (*p < 0.05, ***p < 0.001, vs. MIS VH; # *p* < 0.05, ### *p* < 0.001 vs. Saline VH).

**Fig. 4:**
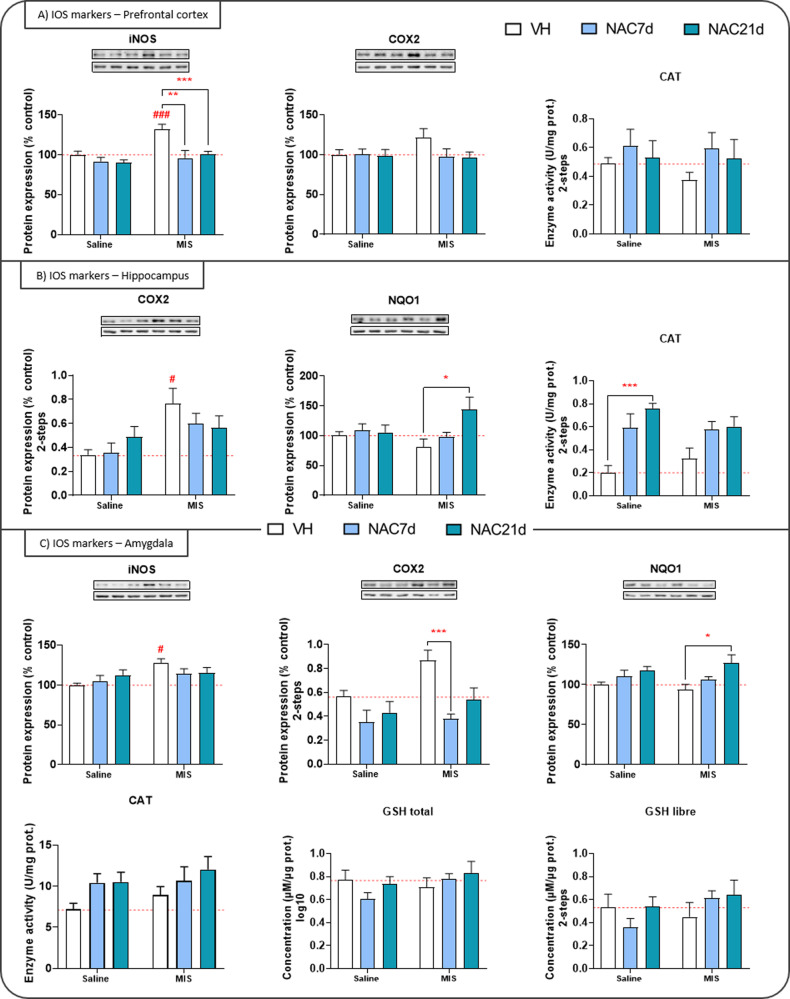
Inflammatory and oxidative stress (IOS) biomarkers in the gestation study. Inflammatory markers expression (iNOS, COX2), antioxidant enzyme activity (GPx, CAT, SOD), and oxidative stress markers (MDA, NRF2, KEAP1, HO1, NQO1, GSHf_ree_, GSH_total_, GSSG) in the prefrontal cortex (**A**), hippocampus (**B**), and amygdala (**C**). Representative bands of iNOS, COX2, KEAP1, HO1, and NQO1 (upper bands) and the loading control, β-actin (lower bands), are shown above their corresponding graph bars. Each column represents mean ± SEM of the protein expression (% control), enzymatic activity (U/mg protein), or concentration (µM/µg protein) of eight animals per group. Two-way ANOVA followed by Bonferroni post-hoc test results: *p < 0.05, **p < 0.01, ***p < 0.001, vs. MIS VH; # *p* < 0.05, ### *p* < 0.001 vs. Saline VH).

Our ex vivo analyses (Figs. 3–4) of brain tissue confirmed the presence of a region-dependent pro-inflammatory/pro-oxidant environment in the MIS-offspring. Importantly, NAC treatments partially and differentially prevented these abnormalities.

### Periadolescence study

In the PFC, the ANOVA analysis showed a significant effect of MIS in iNOS (Fig. 3A) as well as on CAT, MDA, GSH_total_, GSH_free_, and GSSG. Interestingly, there was a great preventive effect of NAC on iNOS and KEAP1 in the MIS-offspring. In the hippocampus (Fig. 3B), no significant effect of MIS was observed, but an effect of NAC was found in COX2 and KEAP1. In the amygdala (Fig. 3C), a significant MIS effect was found in iNOS, COX2, KEAP1, and HO1. Furthermore, NAC significantly reduced COX2 expression while increasing the expression of HO1, NQO1, NRF2, and SOD. In the caudate-putamen, a significant MIS effect was observed in iNOS, GSH_total_, and GSSG, while NAC increased HO1 expression and reduced NRF2 and CAT levels.

#### Gestational study

In the PFC (Fig. 4A), the MIS-offspring exhibited a significant increase in iNOS levels, which were effectively prevented by NAC in both time-windows. Furthermore, NAC21d increased MDA levels and reduced NRF2 levels. In the hippocampus (Fig. 4B), COX2 activity significantly increased due to MIS challenge, although no modulation was observed by NAC. Instead, NAC7d reduced iNOS expression and increased CAT activity, while NAC21d modulated iNOS, CAT, and NQO1. Post-hoc analyses revealed a significant increase in NQO1 activity in MIS-NAC21d animals (*p* < 0.05) and in CAT activity in Saline-NAC21d animals (*p* < 0.01). In the amygdala (Fig. 4C), MIS-challenge led to increased iNOS and COX2 activity and reduced NRF2 expression. NAC7d showed a significant effect in iNOS, COX2, and GSSG, whereas NAC21d modulated iNOS, COX2, NQO1, and CAT. Interestingly, NAC7d prevented the excessive expression of COX2 in the MIS-offspring (*p* < 0.001), thereby reducing its proinflammatory consequences. Additionally, NAC21d increased the levels of the antioxidant biomarker NQO1 (*p* < 0.05). Lastly, in the caudate-putamen, MIS increased COX2 activity and reduced MDA, KEAP1, and GPx. The ANOVA analyses revealed an effect of NAC7d on KEAP1 and NQO1, increasing their levels in MIS-offspring, and an effect of NAC21d modulating COX2, CAT, and GSSG.

## Discussion

This study explores the potential of NAC to prevent the development of schizophrenia-like deficits in the MIS model. NAC treatment was administered during two different time windows: 1) periadolescence, before the onset of initial symptoms, and 2) gestation, aiming to mitigate the risk of neurodevelopmental abnormalities in prenatal offspring associated with heightened IOS. In both cases, NAC successfully prevented certain neuroanatomical, biochemical, and behavioral deficits in the MIS-offspring, with the gestational treatment showing particular efficacy in early intervention. Although several studies have explored the potential therapeutic effects of NAC and other anti-IOS drugs in the MIS model, only a limited number have focused on preventive strategies, particularly during the periadolescent period. Among them, various compounds, such as minocycline [19, 26], omega-3 fatty acids (ω−3) [18], phosphodiesterase-9 inhibitors [27], or even antipsychotic or antidepressant drugs [28, 29] have been administered during periadolescence, with variable result. In this regard, we have recently showed that ω−3 [18] and mangiferin [30] treatments during adolescence were effective in the prevention of behavioral deficits in PPI and some of the volumetric and metabolic deficits seen in this model. Furthermore, we have demonstrated that minocycline-treatment during adolescence partially counteracts volumetric abnormalities and IOS deficits in the MIA model, likely via iNOS and Nrf2–ARE pathways, also increasing the expression of cytoprotective enzymes [31]. On the other hand, the gestational approach has only been explored in two studies. The first demonstrated that NAC treatment during pregnancy was useful in preventing the hippocampal reduction and hippocampal-based memory impairments, along with a partial prevention of gut microbiota deficits observed in MIS-offspring [32]. The second evaluated NAC and omega-3 fatty acids during gestation from the immune insult to the delivery [33]. In contrast to our results, Edemann-Callesen et al. reported only a partial relief in behavior and prevention of microglial activity, as well as enlargement of lateral ventricles in the adult offspring from dams treated with omega-3 fatty acids, evidencing the necessity for further multiscale research. In this line, it is worth noting that there have been no further studies evaluating the effects of NAC administration specifically throughout the entire pregnancy period in this model, including both in vivo and ex vivo techniques, which makes this study of great relevance.

At the biochemical level, we examined the concentration and activity of various markers related to IOS in four brain regions known to be implicated in schizophrenia [7, 34] and affected in the MIS model [18, 19]. Our findings revealed prominent alterations in the PFC and amygdala in the MIS-offspring. Remarkably, NAC treatment demonstrated a preventive effect on these alterations, particularly in the amygdala. The amygdala plays a crucial role in processing fear and threat-related stimuli and has been widely associated with the development of anxiety disorders [35]. Hence, the beneficial antioxidant properties of NAC during pregnancy may contribute to the complete prevention of anxiety-like behaviors observed in the MIS-offspring.

One of the consistently altered markers of IOS in the MIS model is iNOS. This isoform of NOS is known to induce cytotoxicity by promoting the production of nitric oxide (NO) in various cell systems. Activation of iNOS may occur in PolyI:C-treated dams when PolyI:C binds to TLR-3 receptors, subsequently activating the NF-κB pathway [36]. These changes, probably mediated by the interaction between microglia and neurons during neurodevelopment [37], may persist and contribute to the long-term imbalance in IOS observed in this animal model. Furthermore, iNOS has also been implicated in different stages of the disorder [38]. In our study, we observed an increase in iNOS in the MIS-offspring, and importantly, prenatal and periadolescence NAC treatment effectively prevented this alteration.

Moreover, we observed significant differences in the expression of COX2 and SOD in the amygdala during the periadolescence study. The MIS-offspring exhibited increased expression of SOD and decreased expression of COX2, which aligns with the inverse correlation proposed by Lee et al [39].

Additionally, we investigated the antioxidant response element (ARE) proteins, namely HO1 and NQO1, which are known to play a crucial role in cellular antioxidant. Moreover, both HO1 and NQO1 have been reported to be altered in the MIS model [18, 19]. Although we did not observe significant differences in HO1 and NQO1 expression in this model, we did find an impact of NAC treatment on the enzymatic activity of these proteins in the amygdala, particularly when NAC was administered during periadolescence and throughout pregnancy. Similar trends were observed in the PFC, although they did not reach statistical significance. These findings support the notion of an oxidative imbalance within the amygdala and indicate the involvement of HO1 and NQO1 in the preventive effects of NAC. Both proteins are activated through the NRF2 pathway, which involves the dissociation of NRF2 from KEAP1, its translocation into the nucleus, and subsequent binding to target transcription factors [40]. Interestingly, we observed significant differences in KEAP1 levels in MIS-offspring, and these differences were largely prevented by NAC treatments. Nevertheless, we did not find any differences in NRF2 levels. It is possible that the activation of HO1 and NQO1 occur through an alternative pathway that is less commonly observed, potentially explaining this seemingly contradictory results [41]. In any case, our study demonstrates the ability of NAC during periadolescence and, especially, during gestation, to prevent some of the IOS deficits observed in the PFC and amygdala of the MIS model.

At the neuroanatomical level, MIS-offspring did not show the anticipated cortical volume reduction. However, a significant decrease in hippocampal volume was observed in MIS-offspring, and this reduction was effectively prevented by both prenatal NAC treatments, but not by periadolescence treatment. Hippocampal volume reduction is a known characteristic in individuals with schizophrenia and has been associated with various clinical outcomes in this disorder [42]. Interestingly, in our study, this reduction in hippocampal volume was not accompanied by an increase of IOS in this region. This suggests that the symptomatology observed in this animal model may be more closely related to an exacerbated increase in IOS rather than anatomical alterations (i.e., PPI is modulated by the cortico-striatal-pallidum-thalamic (CSPT) circuitry involving the PFC) [43]. Furthermore, MIS-offspring exhibited ventricular enlargement, an alteration that has also been associated with schizophrenia [44] and other neurodevelopmental disorders [45], and has been described in this animal model [17–19]. While periadolescent NAC administration did not lead to a reduction in ventricular volume in MIS animals, a non-significant improvement was observed when the treatment was administered during the final seven days of gestation, indicating that earlier NAC treatment yields more favorable outcomes.

The cerebellum is another structure that may potentially benefit from the administration of NAC. Reduced cerebellar volume has been observed in patients with schizophrenia, and these alterations have been associated with the onset of positive symptoms [46], although it is worth noting that this reduction has also been attributed to the side effects of antipsychotics [17]. In our study, although NAC treatment during periadolescence did not have an impact on cerebellar volume, the administration of NAC throughout gestation nearly counteracted this reduction. These findings suggest that the prenatal approach of NAC treatment may have some preventive potential in addressing cerebellar-related deficits in schizophrenia.

Regarding white matter changes, we found a reduction in corpus callosum volume in MIS-offspring, which is consistent with previous studies demonstrating a generalized decrease in white matter volume [47] in individuals with schizophrenia. This alteration is associated with impaired connectivity between the two cerebral hemispheres. Interestingly, while NAC treatment during periadolescence did not prevent this deficit in MIS-offspring, both prenatal treatments completely prevented the reduction in corpus callosum volume. In addition to assessing volumetric changes, we also examined white matter alterations in the corpus callosum, PFC, and hippocampus using DTI. Surprisingly, we detected a significant increase in FA in the corpus callosum, and this increase was fully prevented by both NAC treatments during pregnancy. These findings contrast with clinical reports indicating reduced FA and MD in patients with schizophrenia and adolescents with early-onset psychosis [48]. Currently, we do not have an explanation for this unexpected finding. One possibility could be related to fiber compaction in the corpus callosum, leading to volume reduction and subsequently an increase in FA in the MIS model. Nevertheless, it is important to note that this increase in FA does not correlate with the corpus callosum size, as we observed the same volume reduction in both the animal model and the human disorder.

In terms of brain metabolic changes, abnormalities in the levels of glutamate + glutamine (neurotransmitter and precursor) and N-Acetyl Aspartate + glutamic acid (NAA + NAAG; neuronal viability) in the PFC have been associated with schizophrenia in patients with psychotic disorders and individuals at ultra-high risk for psychosis [49]. These abnormalities have also been implicated in the progression of the pathology [50]. In the MIS model, our study is the first report to tackle this issue. We did not observe significant differences in metabolite concentrations following MIS challenge. However, there was a trend towards increased NAA + NAAG and choline levels in the Saline and MIS-offspring treated with NAC during pregnancy. This potential increase in NAA + NAAG levels is particularly interesting and promising, as reduced NAA levels have been associated with poor disease progression in schizophrenia [50].

In terms of behavioral tests, we were able to confirm the presence of a deficit in PPI and increased anxiety levels in MIS-offspring, which is consistent with findings from other studies [51, 52]. Importantly, all NAC treatments, particularly the prenatal approaches, effectively prevented these deficits in both PPI and anxiety. Deficits in PPI and anxiety are well-documented in clinical studies [53] and preclinical research [54]. Specifically, PPI deficits have been associated with impairment in the PFC [55], which was also altered in terms of IOS in our study. Furthermore, the cerebellum, another region affected in our MIS animals, has been implicated in auditory processing impairment [56], which could be involved in the observed deficits in response to acoustic stimuli in this behavioral test.

Overall, the three NAC treatment approaches effectively prevented behavioral deficits in sensorimotor gating and anxiety, as well as some of the biochemical alterations in the brain. However, the impact on neuroanatomical anomalies was not consistent across the different approaches. Treatment with NAC during periadolescence had limited effects on brain anatomy, whereas administration during pregnancy showed more promising results, possibly due to its ability to intervene earlier in the developmental process. It is worth noting that neuroanatomical abnormalities have been reported prior to the onset of symptoms in schizophrenia [57]. Therefore, initiating therapeutic interventions with a safe compound during pregnancy may have the potential to prevent long-term deficits associated with IOS dysfunction in offspring, by addressing neurodevelopmental abnormalities at an early stage.

This study has several limitations that should be acknowledged. Firstly, the study was conducted exclusively in male-offspring. It is known that the effects of MIS differ between males and females in terms of functional and morphological changes [58]. Additionally, the onset of schizophrenia symptoms occurs later in women than in men. However, exploring these gender differences was beyond the scope of our current study, and focusing on male offspring allowed us to establish common therapeutic and sampling windows. Nonetheless, it is important to extend our approaches to include female offspring in future investigations. Secondly, a modification to the acquisition protocol in the MRS study, such as using a larger voxel size and increasing the number of repetitions, could have potentially yielded stronger and more robust results by improving the signal-to-noise ratio. Thirdly, it should be noted that the periadolescence approach did not include studies on anxiety, DTI, and MRS, as these aspects were incorporated into the gestational approach based on the promising results obtained during periadolescence, This expansion of the experimental battery allowed for a more comprehensive assessment of the model. Fourthly, despite the sample size of each group seems to be adequate for the purposes of this study, we acknowledge that MRI volumetric results could have benefited from a limited increase in the number of animals included in each group. Finally, we must acknowledge that no corrections for multiple comparisons were performed. This decision is justified by the exploratory nature of the study and by the fact that most measurements were decided a priori, based on the results of previous studies. In addition, these methods assume independency of the tests, which may not be true in our complex dataset due to potential interdependencies between markers and brain regions examined, which could potentially blur true associations or patterns. However, we recognize the inherent bias associated with this approach that may lead to a certain degree of Type I error. Consequently, interpretation of these results should be taken with caution and emphasize the need for replication studies to further validate these findings.

In summary, treatment with NAC during both gestation and periadolescence effectively prevented behavioral deficits and various biochemical alterations in the MIS model. Additionally, prenatal administration of NAC demonstrated the ability to prevent some of the neuroanatomical anomalies observed in the MIS model. These findings suggest that early therapeutic interventions hold promise in reducing the prenatal IOS status and decreasing the severity and risk of developing schizophrenia-like deficits in offspring.

### Supplementary information

Supplementary Figure 1

## Data Availability

The dataset generated and analyzed during the current study are available from the corresponding author on reasonable request.

## References

[1] Disease GBD, Injury I, Prevalence C. (2018). Global, regional, and national incidence, prevalence, and years lived with disability for 354 diseases and injuries for 195 countries and territories, 1990-2017: a systematic analysis for the Global Burden of Disease Study 2017. Lancet.

[2] Tandon N, Shah J, Keshavan MS, Tandon R (2012). Attenuated psychosis and the schizophrenia prodrome: current status of risk identification and psychosis prevention. Neuropsychiatry (Lond).

[3] Pasternak O, Kubicki M, Shenton ME. In vivo imaging of neuroinflammation in schizophrenia. Schizophr Res. 2015. 10.1016/j.schres.2015.05.034 PMC4668243 26048294

[4] Kirkpatrick B, Miller BJ (2013). Inflammation and schizophrenia. Schizophr Bull.

[5] Martinez-Cengotitabengoa M, Mico JA, Arango C, Castro-Fornieles J, Graell M, Paya B (2014). Basal low antioxidant capacity correlates with cognitive deficits in early onset psychosis. A 2-year follow-up study. Schizophr Res.

[6] Meyer U (2013). Developmental neuroinflammation and schizophrenia. Prog Neuro-Psychopharmacol Biol psychiatry.

[7] Selemon LD, Zecevic N (2015). Schizophrenia: a tale of two critical periods for prefrontal cortical development. Transl psychiatry.

[8] Marin O (2016). Developmental timing and critical windows for the treatment of psychiatric disorders. Nat Med.

[9] Studerus E, Ramyead A, Riecher-Rossler A (2017). Prediction of transition to psychosis in patients with a clinical high risk for psychosis: a systematic review of methodology and reporting. Psychol Med.

[10] Andrade C. Antipsychotic Augmentation With N-Acetylcysteine for Patients With Schizophrenia. J Clin Psychiatry 2022;83. 10.4088/JCP.22f14664 36170201

[11] Bradlow RCJ, Berk M, Kalivas PW, Back SE, Kanaan RA (2022). The Potential of N-Acetyl-L-Cysteine (NAC) in the Treatment of Psychiatric Disorders. CNS drugs.

[12] Minarini A, Ferrari S, Galletti M, Giambalvo N, Perrone D, Rioli G (2017). N-acetylcysteine in the treatment of psychiatric disorders: current status and future prospects. Expert Opin Drug Metab Toxicol.

[13] Firth J, Teasdale SB, Allott K, Siskind D, Marx W, Cotter J (2019). The efficacy and safety of nutrient supplements in the treatment of mental disorders: a meta-review of meta-analyses of randomized controlled trials. World Psychiatry.

[14] Monte AS, da Silva FER, Lima CNC, Vasconcelos GS, Gomes NS, Miyajima F (2020). Sex influences in the preventive effects of N-acetylcysteine in a two-hit animal model of schizophrenia. J Psychopharmacol.

[15] Cabungcal JH, Counotte DS, Lewis E, Tejeda HA, Piantadosi P, Pollock C (2014). Juvenile antioxidant treatment prevents adult deficits in a developmental model of schizophrenia. Neuron.

[16] Swanepoel T, Moller M, Harvey BH (2018). N-acetyl cysteine reverses bio-behavioural changes induced by prenatal inflammation, adolescent methamphetamine exposure and combined challenges. Psychopharmacology.

[17] Casquero-Veiga M, Garcia-Garcia D, MacDowell KS, Perez-Caballero L, Torres-Sanchez S, Fraguas D (2019). Risperidone administered during adolescence induced metabolic, anatomical and inflammatory/oxidative changes in adult brain: A PET and MRI study in the maternal immune stimulation animal model. Eur Neuropsychopharmacol : J Eur Coll Neuropsychopharmacol.

[18] Casquero-Veiga M, Romero-Miguel D, MacDowell KS, Torres-Sanchez S, Garcia-Partida JA, Lamanna-Rama N (2021). Omega-3 fatty acids during adolescence prevent schizophrenia-related behavioural deficits: Neurophysiological evidences from the prenatal viral infection with PolyI:C. Eur Neuropsychopharmacol : J Eur Coll Neuropsychopharmacol.

[19] Romero-Miguel D, Casquero-Veiga M, MacDowell KS, Torres-Sanchez S, Garcia-Partida JA, Lamanna-Rama N (2021). A Characterization of the Effects of Minocycline Treatment During Adolescence on Structural, Metabolic, and Oxidative Stress Parameters in a Maternal Immune Stimulation Model of Neurodevelopmental Brain Disorders. Int J Neuropsychopharmacol.

[20] Percie du Sert N, Hurst V, Ahluwalia A, Alam S, Avey MT, Baker M (2020). The ARRIVE guidelines 2.0: Updated guidelines for reporting animal research. PLoS Biol.

[21] Paxinos G, Watson C. *The rat brain in stereotaxic coordinates*. 4th edn. Academic Press San Diego, 2008.

[22] Sargolzaei S, Cai Y, Wolahan SM, Gaonkar B, Sargolzaei A, Giza CC (2018). A Comparative Study of Automatic Approaches for Preclinical MRI-based Brain Segmentation in the Developing Rat. Annu Int Conf IEEE Eng Med Biol Soc IEEE Eng Med Biol Soc Annu Int Conf.

[23] Provencher SW (2001). Automatic quantitation of localized in vivo 1H spectra with LCModel. NMR Biomed.

[24] MacDowell KS, Garcia-Bueno B, Madrigal JL, Parellada M, Arango C, Mico JA (2013). Risperidone normalizes increased inflammatory parameters and restores anti-inflammatory pathways in a model of neuroinflammation. Int J Neuropsychopharmacol.

[25] Das NP, Ratty AK (1987). Studies on the effects of the narcotic alkaloids, cocaine, morphine, and codeine on nonenzymatic lipid peroxidation in rat brain mitochondria. Biochem Med Metab Biol.

[26] Giovanoli S, Engler H, Engler A, Richetto J, Feldon J, Riva MA (2016). Preventive effects of minocycline in a neurodevelopmental two-hit model with relevance to schizophrenia. Transl Psychiatry.

[27] Scarborough J, Mattei D, Dorner-Ciossek C, Sand M, Arban R, Rosenbrock H (2021). Symptomatic and preventive effects of the novel phosphodiesterase-9 inhibitor BI 409306 in an immune-mediated model of neurodevelopmental disorders. Neuropsychopharmacol : Off Publ Am Coll Neuropsychopharmacol.

[28] Piontkewitz Y, Arad M, Weiner I (2011). Risperidone administered during asymptomatic period of adolescence prevents the emergence of brain structural pathology and behavioral abnormalities in an animal model of schizophrenia. Schizophr Bull.

[29] Meyer U, Spoerri E, Yee BK, Schwarz MJ, Feldon J (2010). Evaluating early preventive antipsychotic and antidepressant drug treatment in an infection-based neurodevelopmental mouse model of schizophrenia. Schizophr Bull.

[30] Garcia-Partida JA, Torres-Sanchez S, MacDowell K, Fernandez-Ponce MT, Casas L, Mantell C (2022). The effects of mango leaf extract during adolescence and adulthood in a rat model of schizophrenia. Front Pharmacol.

[31] Romero-Miguel D, Casquero-Veiga M, MacDowell KS, Torres-Sanchez S, Garcia-Partida JA, Lamanna-Rama N et al. A characterization of the effects of minocycline treatment during adolescence on structural, metabolic and oxidative stress parameters in a maternal immune stimulation model of neurodevelopmental brain disorders. Int J Neuropsychopharm. 2021. 10.1093/ijnp/pyab036 PMC8453277 34165516

[32] Romero-Miguel D, Casquero-Veiga M, Fernandez J, Lamanna-Rama N, Gomez-Rangel V, Galvez-Robleno C et al. Maternal Supplementation with N-Acetylcysteine Modulates the Microbiota-Gut-Brain Axis in Offspring of the Poly I:C Rat Model of Schizophrenia. *Antioxidants* 2023;12. 10.3390/antiox12040970 PMC10136134 37107344

[33] Edemann-Callesen H, Bernhardt N, Hlusicka EB, Hintz F, Habelt B, Winter R et al. Supplement Treatment with NAC and Omega-3 Polyunsaturated Fatty Acids during Pregnancy Partially Prevents Schizophrenia-Related Outcomes in the Poly I:C Rat Model. Antioxidants 2023;12. 10.3390/antiox12051068 PMC10215079 37237933

[34] Bartholomeusz CF, Cropley VL, Wannan C, Di Biase M, McGorry PD, Pantelis C (2017). Structural neuroimaging across early-stage psychosis: Aberrations in neurobiological trajectories and implications for the staging model. Aust NZ J Psychiatry.

[35] Sah P (2017). Fear, Anxiety, and the Amygdala. Neuron.

[36] Yang CS, Kim JJ, Lee SJ, Hwang JH, Lee CH, Lee MS (2013). TLR3-triggered reactive oxygen species contribute to inflammatory responses by activating signal transducer and activator of transcription-1. J Immunol.

[37] Chamera K, Trojan E, Kotarska K, Szuster-Gluszczak M, Bryniarska N, Tylek K et al. Role of Polyinosinic:Polycytidylic Acid-Induced Maternal Immune Activation and Subsequent Immune Challenge in the Behaviour and Microglial Cell Trajectory in Adult Offspring: A Study of the Neurodevelopmental Model of Schizophrenia. Int J Mol Sci. 2021;22. 10.3390/ijms22041558 PMC7913889 33557113

[38] Garcia-Bueno B, Bioque M, Mac-Dowell KS, Barcones MF, Martinez-Cengotitabengoa M, Pina-Camacho L (2014). Pro-/anti-inflammatory dysregulation in patients with first episode of psychosis: toward an integrative inflammatory hypothesis of schizophrenia. Schizophr Bull.

[39] Lee JA, Song HY, Ju SM, Lee SJ, Kwon HJ, Eum WS (2009). Differential regulation of inducible nitric oxide synthase and cyclooxygenase-2 expression by superoxide dismutase in lipopolysaccharide stimulated RAW 264.7 cells. Exp Mol Med.

[40] Loboda A, Damulewicz M, Pyza E, Jozkowicz A, Dulak J (2016). Role of Nrf2/HO-1 system in development, oxidative stress response and diseases: an evolutionarily conserved mechanism. Cell Mol Life Sci : CMLS.

[41] Zhang A, Suzuki T, Adachi S, Naganuma E, Suzuki N, Hosoya T (2021). Distinct Regulations of HO-1 Gene Expression for Stress Response and Substrate Induction. Mol Cell Biol.

[42] Brosch K, Stein F, Schmitt S, Pfarr JK, Ringwald KG, Thomas-Odenthal F (2022). Reduced hippocampal gray matter volume is a common feature of patients with major depression, bipolar disorder, and schizophrenia spectrum disorders. Mol Psychiatry.

[43] Murray AJ, Rogers JC, Katshu M, Liddle PF, Upthegrove R (2021). Oxidative Stress and the Pathophysiology and Symptom Profile of Schizophrenia Spectrum Disorders. Front Psychiatry.

[44] Liu C, Kim WS, Shen J, Tsogt U, Kang NI, Lee KH (2022). Altered Neuroanatomical Signatures of Patients With Treatment-Resistant Schizophrenia Compared to Patients With Early-Stage Schizophrenia and Healthy Controls. Front Psychiatry.

[45] Shi J, Guo H, Liu S, Xue W, Fan F, Li H (2021). Subcortical Brain Volumes Relate to Neurocognition in First-Episode Schizophrenia, Bipolar Disorder, Major Depression Disorder, and Healthy Controls. Front Psychiatry.

[46] Laidi C, d’Albis MA, Wessa M, Linke J, Phillips ML, Delavest M (2015). Cerebellar volume in schizophrenia and bipolar I disorder with and without psychotic features. Acta Psychiatr Scand.

[47] Cetin-Karayumak S, Di Biase MA, Chunga N, Reid B, Somes N, Lyall AE (2020). White matter abnormalities across the lifespan of schizophrenia: a harmonized multi-site diffusion MRI study. Mol Psychiatry.

[48] Barth C, Kelly S, Nerland S, Jahanshad N, Alloza C, Ambrogi S et al. In vivo white matter microstructure in adolescents with early-onset psychosis: a multi-site mega-analysis. Mol Psychiatry 2022. 10.1038/s41380-022-01901-3 PMC10005938 36510004

[49] Liemburg E, Sibeijn-Kuiper A, Bais L, Pijnenborg G, Knegtering H, van der Velde J (2016). Prefrontal NAA and Glx Levels in Different Stages of Psychotic Disorders: a 3T 1H-MRS Study. Sci Rep.

[50] Whitehurst TS, Osugo M, Townsend L, Shatalina E, Vava R, Onwordi EC (2020). Proton Magnetic Resonance Spectroscopy of N-acetyl Aspartate in Chronic Schizophrenia, First Episode of Psychosis and High-Risk of Psychosis: A Systematic Review and Meta-Analysis. Neurosci Biobehav Rev.

[51] Quagliato LA, de Matos U, Nardi AE (2021). Maternal immune activation generates anxiety in offspring: A translational meta-analysis. Transl Psychiatry.

[52] Hadar R, Soto-Montenegro ML, Gotz T, Wieske F, Sohr R, Desco M (2015). Using a maternal immune stimulation model of schizophrenia to study behavioral and neurobiological alterations over the developmental course. Schizophr Res.

[53] Mena A, Ruiz-Salas JC, Puentes A, Dorado I, Ruiz-Veguilla M, De la Casa LG (2016). Reduced Prepulse Inhibition as a Biomarker of Schizophrenia. Front Behav Neurosci.

[54] Powell SB, Zhou X, Geyer MA (2009). Prepulse inhibition and genetic mouse models of schizophrenia. Behav Brain Res.

[55] Miller EA, Kastner DB, Grzybowski MN, Dwinell MR, Geurts AM, Frank LM (2021). Robust and replicable measurement for prepulse inhibition of the acoustic startle response. Mol Psychiatry.

[56] Andreasen NC, Pierson R (2008). The role of the cerebellum in schizophrenia. Biol Psychiatry.

[57] DeLisi LE (2008). The concept of progressive brain change in schizophrenia: implications for understanding schizophrenia. Schizophr Bull.

[58] Casquero-Veiga M, Lamanna-Rama N, Romero-Miguel D, Rojas-Marquez H, Alcaide J, Beltran M (2022). The Poly I:C maternal immune stimulation model shows unique patterns of brain metabolism, morphometry, and plasticity in female rats. Front Behav Neurosci.

