# Supplementary figures and images for: N-acetylcysteine during critical neurodevelopmental periods prevents behavioral and neurochemical deficits in the Poly I:C rat model of schizophrenia
Source: Transl Psychiatry. 2024 Jan 8;14:14. doi: 10.1038/s41398-023-02652-7 (PMC10774365; doi:10.1038/s41398-023-02652-7)

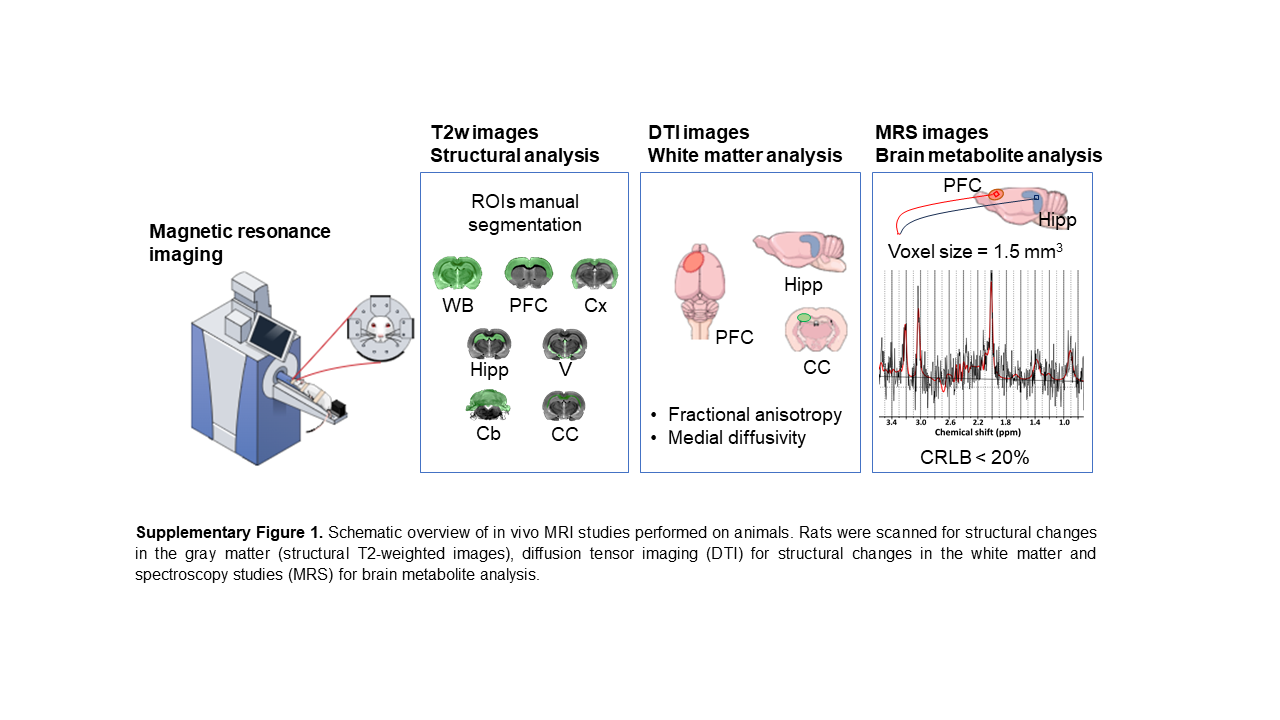

Supplement: Supplementary file 1 — Supplementary Figure 1 [file 41398_2023_2652_MOESM1_ESM.tif]
